# Targeting SQLE-Mediated Cholesterol Metabolism to Promote Oxidative Stress and Attenuate Drug Resistance in Osteosarcoma

**DOI:** 10.3390/antiox15091122

**Published:** 2026-09-04

**Authors:** Amonnat Sukhamwang, Dumnoensun Pruksakorn, Pornngarm Dejkriengkraikul, Michael A. Dengler, Supachai Yodkeeree

**Affiliations:** 1 Department of Biochemistry, Faculty of Medicine, Chiang Mai University, Chiang Mai 50200, Thailand; amonnat_s@cmu.ac.th (A.S.); pornngarm.d@cmu.ac.th (P.D.); 2 Center of Multidisciplinary Technology for Advanced Medicine (CMUTEAM), Faculty of Medicine, Chiang Mai University, Chiang Mai 50200, Thailand; dumnoensun.p@cmu.ac.th; 3 Musculoskeletal Science and Translational Research (MSTR) Center, Faculty of Medicine, Chiang Mai University, Chiang Mai 50200, Thailand; 4 Department of Orthopedics, Faculty of Medicine, Chiang Mai University, Chiang Mai 50200, Thailand; 5 Anticarcinogenesis and Apoptosis Research Cluster, Faculty of Medicine, Chiang Mai University, Chiang Mai 50200, Thailand; 6 Division of Oncology, Department of Internal Medicine, Medical University of Graz, Auenbruggerplatz 15, 8036 Graz, Austria; michael.dengler@medunigraz.at

**Keywords:** osteosarcoma, transcriptome, chemotherapy resistance, cholesterol biosynthesis, SQLE

## Abstract

High-grade osteosarcoma presents a significant clinical challenge due to unpredictable therapeutic responses and aggressive progression. This study aimed to identify the critical molecular pathways driving chemotherapy resistance and aggressive phenotypes in osteosarcoma patients. Through transcriptomic and bioinformatic analyses, we identified cholesterol biosynthesis as a key upregulated metabolic pathway in poor chemotherapy responders, where squalene epoxidase (*SQLE*) emerged as an exploratory candidate hub gene whose elevated expression significantly correlates with shortened survival in the TCGA cohort. We validated these findings by administering terbinafine, a known SQLE inhibitor. In highly chemoresistant SaOS-2 cells exhibiting the highest baseline *SQLE* expression, terbinafine synergistically sensitized cells to doxorubicin by driving cell death partly through apoptosis, as confirmed by caspase inhibition. The combination also promoted ferroptosis, indicated by elevated ROS and MDA along with decreased FSP1 and GPX4 expression. Furthermore, the co-treatment effectively suppressed clonogenic potential, induced G2/M phase cell cycle arrest, and inhibited metastatic progression. These effects were mediated by the modulation of cell proliferation, metastasis, and survival genes through the coordinated regulation of the PI3K/AKT/mTOR, ERK, and JNK signaling cascades. Together, these results highlight the therapeutic potential of targeting the SQLE pathway to overcome doxorubicin resistance and suppress aggressive progression in high-grade osteosarcoma.

## 1. Introduction

Osteosarcoma is the most common primary malignant bone tumor, accounting for approximately 20% of all bone cancers [1], with a global incidence estimated at 3–4 cases per million annually, predominantly affecting adolescents and young adults [2]. While the prognosis for patients with localized disease has improved, achieving a 5-year survival rate of 60–70%, this rate drops significantly in cases of metastatic or recurrent disease, emphasizing the urgent need for more effective therapeutic strategies [3]. The standard approach, comprising surgical resection and the MAP (methotrexate, doxorubicin, and cisplatin) chemotherapy regimen, has been foundational in patient management [4].

However, chemoresistance remains a major obstacle, driven by multidimensional biological pathways that enable tumor cell survival and adaptation under chemotherapeutic stress [5]. For instance, the overexpression of drug efflux transporters, such as ATP-binding cassette subfamily B member 1 (ABCB1), facilitates the expulsion of chemotherapeutic agents from cells [6]. In osteosarcoma, ABCB1 promotes doxorubicin resistance by limiting intracellular drug accumulation, thereby reducing cytotoxic efficacy. Moreover, the co-occurrence of elevated ABCB1 and suppressed ABCA1 expression serves as a molecular signature for poor chemotherapy response [7]. Furthermore, enhanced DNA repair pathways contribute to cisplatin resistance in osteosarcoma, particularly through the positive ERCC1 expression, which is a vital component of the nucleotide excision repair (NER) machinery [8]. Beyond these factors, metabolic reprogramming, involving glycolytic pathways, pentose phosphate pathways, and altered fatty acid metabolism, has been increasingly recognized as a driver of cell proliferation, metastasis, and drug resistance in osteosarcoma [9].

The clinical prognosis is particularly poor for patients presenting with metastatic disease at diagnosis. Previous studies reported that such patients treated with standard MAP chemotherapy achieved only a 21% two-year event-free survival (EFS) and 55% overall survival (OS), significantly lower than the 75% EFS and 94% OS observed in non-metastatic patients [10]. Even with alternative regimens, such as the non-high-dose methotrexate-based OGS-12 protocol, survival rates for metastatic patients remain suboptimal, with four-year EFS and OS rates of 24% and 27%, respectively [11].

Predicting chemotherapy response prior to the initiation of neoadjuvant therapy would significantly benefit patients by enabling the selection of personalized treatment strategies. In this study, we aimed to identify predictive biomarkers for chemotherapy response in high-grade osteosarcoma. To achieve this, we performed transcriptomic profiling on treatment-naïve tumor tissues collected at initial diagnosis. Samples were categorized into ‘good responders’ (≥90% tumor necrosis) and ‘poor responders’ (≤60% tumor necrosis, all of whom subsequently displayed metastatic progression during follow-up) based on their subsequent clinical outcomes following chemotherapy. Subsequently, we validated these clinical findings through in vitro functional assays to elucidate the underlying molecular mechanisms.

## 2. Materials and Methods

### 2.1. Reagents and Chemicals

Dulbecco’s modified Eagle’s medium (DMEM), penicillin/streptomycin, and trypsin-EDTA were obtained from Gibco (Grand Island, NY, USA), while fetal bovine serum (FBS) was provided by Hyclone (Logan, UT, USA). An FITC Annexin V kit was obtained from Elabscience Biotechnology (Houston, TX, USA). MitoView^TM^ 633 was obtained from Biotium (Fremont, CA, USA). Antibodies specific to c-FLIP, c-IAP2, BCL-2, BCL-XL, p-GSK3, GSK3, p-ERK, ERK, N-cadherin, vimentin, cleaved caspase-3, cyclin-B, and β-actin were obtained from Cell Signaling Technology (Danvers, MA, USA), whereas antibodies specific to MT1MMP, p-mTOR, mTOR, p-AKT, AKT, PARP-1, GPX-4, FSP-1, CDC25C, and p21 were purchased from Abclonal (Woburn, MA, USA). ECL reagent was supplied by Bio-Helix (New Taipei, Taiwan). Matrigel was obtained from Becton Dickinson (Bedford, MA, USA). Cisplatin was obtained from Sigma (St. Louis, MO, USA). Doxorubicin hydrochloride salt was obtained from LC Laboratories (Woburn, MA, USA). Terbinafine, Squalene, and Z-VAD-FMK were obtained from MedChemExpress (Monmouth Junction, NJ, USA).

### 2.2. mRNA Sequencing Data Analysis

Transcriptomic data was obtained from our previous study [12]. Briefly, treatment-naïve biopsy specimens were collected prior to neoadjuvant chemotherapy, and patient chemoresponse was subsequently evaluated based on post-treatment histological tumor necrosis percentage (≥90% for good responders and ≤60% for poor responders) [12]. Notably, all poor responders in this cohort also subsequently exhibited metastatic progression during follow-up, reflecting an intrinsically aggressive phenotype (detailed patient-level clinical characteristics are provided in Supplementary Table S2). To identify differentially expressed genes (DEGs) relevant to chemotherapy response, we re-analyzed the raw data using updated criteria. Multiple-testing adjustment was performed using the Benjamini–Hochberg False Discovery Rate (FDR) procedure (p_adj_) within DESeq2. DEGs were identified using a stringent cutoff of |log_2_FC| ≥ 1.4 and p_adj_ < 0.05. In addition, genes meeting an exploratory nominal threshold of unadjusted *p*-value < 0.05 and |log_2_FC| ≥ 1.4 were classified as the nominal exploratory DEG set, which was subsequently utilized for functional enrichment, pathway mapping, and PPI network analysis. The volcano plot was created using the EnhancedVolcano R package (v1.10.0).

### 2.3. Protein–Protein Interaction and Identification of Hub Genes and Subnetwork Analysis

The protein–protein interaction (PPI) networks were built by Cytoscape software (v3.10.4). To identify highly connected gene clusters within the network, the Molecular Complex Detection (MCODE v2.0.3) app from Cytoscape was applied. Subsequently, functional enrichment analysis of the identified modules was performed using the ClueGO (v2.5.10) app in Cytoscape to build and compare networks of functionally related Gene Ontology (GO) terms.

### 2.4. Determination of Gene Expressions by RT-qPCR Analysis

Total RNA was extracted from osteosarcoma cell lines using NucleoZOL reagent (Macherey-Nagel™, Düren, Germany) according to the manufacturer’s instructions. RNA concentration and purity were determined using a NanoDrop 2000/2000c spectrophotometer (Thermo Fisher Scientific, Waltham, MA, USA). Complementary DNA (cDNA) was synthesized from total RNA using the ReverTra Ace™ qPCR RT Master Mix with gDNA Remover (TOYOBO, Osaka, Japan). Quantitative real-time PCR (RT-qPCR) was performed using the THUNDERBIRD™ Next SYBR qPCR Mix (TOYOBO, Osaka, Japan) on an Applied Biosystems QuantStudio 6 Flex system (software v.1.0, Waltham, MA, USA). Gene expression levels were calculated using the 2^-ΔΔCT^ method, with *GAPDH* used as the internal normalization control.

### 2.5. The Correlation of SQLE Expression with Overall Survival, Disease-Specific Survival, and Disease-Free Survival in Sarcoma Samples

Survival analysis concerning *SQLE* mRNA expression was performed using the TCGA PanCancer Atlas Sarcoma cohort via the cBioPortal for Cancer Genomics platform. The prognostic impact of *SQLE* expression on overall survival (OS), disease-specific survival (DSS), and disease-free survival (DFS) was evaluated. Kaplan–Meier survival curves were generated, and statistical significance was determined to assess the prognostic value of *SQLE* expression in sarcoma tissues.

### 2.6. Cell Cultures

Osteosarcoma cell lines were sourced as follows: 143B (CRL-8303) and SaOS-2 (HTB-85) from ATCC (Manassas, VA, USA), and U2OS (CLS-300364) from Cell Lines Service (GmbH, Eppelheim, Germany). Cells were maintained in DMEM containing 10% fetal bovine serum (FBS) and 1% penicillin-streptomycin at 37 °C in a humidified incubator with 5% CO_2_.

### 2.7. Chemo-Sensitivity Testing by MTT Colorimetric Assay

Cell sensitivity to cisplatin and doxorubicin was assessed using the 3-(4,5-dimethylthiazol-2-yl)-2,5-diphenyltetrazolium bromide (MTT) assay. Briefly, SaOS-2, U2OS, and 143B cells were seeded into 96-well plates at densities of 3 × 10^3^, 2.5 × 10^3^, and 7.5 × 10^2^ cells/well, respectively. After incubation, cells were treated with varying concentrations of cisplatin (0–25 μM) or doxorubicin (0–1000 nM) for 72 h. Subsequently, 10 μL of 3-(4,5-dimethylthiazol-2-yl)-2,5-diphenyltetrazolium bromide (MTT) solution (0.5 mg/mL in phosphate-buffered saline (PBS)) was added to each well, and the plates were incubated for 4 h. Following the removal of the culture medium, the resulting formazan crystals were dissolved in 200 μL of DMSO. Absorbance was measured at a primary wavelength of 540 nm and a reference wavelength of 630 nm using a UV-visible spectrophotometer. All assays were performed in triplicate, and cell viability was expressed as a percentage relative to the untreated control group. To evaluate the synergistic effects of drug combinations, SaOS-2 and 143B cells were co-treated with varying concentrations of cisplatin or doxorubicin in the presence or absence of terbinafine (0–75 μM). Then, cell viability was assessed using the MTT assay, as previously described. Furthermore, to investigate the underlying mechanisms of terbinafine-mediated chemosensitization, SaOS-2 cells were seeded at a density of 1.5× 10^4^ cells/well in 96-well plates. Cells were treated with 150 nM doxorubicin and 75 μM terbinafine for 48 h in the presence or absence of either 50 μM Z-VAD-FMK (a pan-caspase inhibitor to assess apoptotic contribution) or 250 μM squalene (to determine the involvement of squalene accumulation in drug synergism). Cell viability for all conditions was subsequently assessed using the MTT assay, as described above.

### 2.8. Apoptosis Assay

Apoptosis and necrosis were evaluated using the Annexin V-FITC/PI Apoptosis Detection Kit (Elabscience Biotechnology Inc., Houston, TX, USA) according to the manufacturer’s instructions. SaOS-2 cells were treated with 150 nM doxorubicin in the presence or absence of terbinafine (50 or 75 μM) for 48 h. Following treatment, cells were harvested using trypsin, rinsed with PBS, and stained with 2.5 μL of Annexin V-FITC and 2.5 μL of propidium iodide (PI) at room temperature for 15 min. The stained cells were analyzed via flow cytometry, and data processing was performed using CytExpert for DxFLEX 2.0 software.

### 2.9. Mitochondrial Membrane Potential

Mitochondrial membrane potential was assessed using the MitoView™ 633 dye (Biotium, Fremont, CA, USA) according to the manufacturer’s protocol. Briefly, SaOS-2 cells were treated with 150 nM doxorubicin in the presence or absence of terbinafine (50 or 75 μM) for 48 h. Following treatment, cells were harvested by trypsinization and incubated with 100 nM MitoView™ 633 in incomplete DMEM for 20 min at 37 °C in a humidified 5% CO_2_ atmosphere, protected from light. Cells were subsequently washed with PBS, and fluorescence was measured via flow cytometry (excitation/emission: 638/660 nm). Data analysis was performed using CytExpert for DxFLEX software.

### 2.10. Dichloro-Dihydro-Fluorescein Diacetate (DCFH-DA) Assay

Intracellular reactive oxygen species (ROS) levels were measured using the 2′,7′-dichlorodihydrofluorescein diacetate (DCF-DA) assay. Briefly, SaOS-2 cells (8 × 10^3^ cells/well) were seeded in 96-well plates and treated with 150 nM doxorubicin in the presence or absence of terbinafine (50 or 75 μM) for 24 h. Following treatment, cells were stained with 10 μM DCF-DA for 45 min at 37 °C. After a single wash with PBS, fluorescence intensity was measured kinetically at excitation and emission wavelengths of 485 nm and 535 nm, respectively, using a fluorescence microplate reader.

### 2.11. Thiobarbituric Acid Reactive Substances (TBARS) Assay

SaOS-2 cells were seeded in 6-well plates (8.5 × 10^5^) and treated with 150 nM doxorubicin in the presence or absence of terbinafine (50 or 75 μM) for 1 h. Following treatment, cells were washed twice with PBS and harvested using 250 μL of lysis buffer per well. The assay was performed as follows: 100 μL of each lysate sample or standard was transferred to a 1.5 mL centrifuge tube with a secure-lock or screw cap. Subsequently, 100 μL of 8.0% (*w*/*v*) SDS solution was added, followed by 800 μL of the color reagent. The color reagent was prepared by mixing 106 mg of thiobarbituric acid (TBA) with 10 mL of acetic acid solution (prepared by diluting 10 mL of concentrated acetic acid in 40 mL of DI water) and 10 mL of 3.5 M NaOH. The tubes were tightly sealed and heated at 95 °C for 1 h in a dry bath. The reaction was terminated by cooling the tubes in an ice bath, after which 450 μL of butanol was added. Following a 5 min incubation on ice, the tubes were centrifuged at 1600× *g* for 10 min at room temperature. Finally, 200 μL of the supernatant was transferred to a plate, ensuring the pellet remained undisturbed. Fluorescence was recorded at an excitation wavelength of 530 nm and an emission wavelength of 556 nm (bandwidth ≤ 10 nm).

### 2.12. PI Staining

Cell cycle distribution was analyzed using propidium iodide (PI) staining followed by flow cytometry. SaOS-2 cells were treated with various concentrations of doxorubicin, in the presence or absence of terbinafine, for 48 h. Following treatment, cells were harvested and fixed with ice-cold 70% (*v*/*v*) ethanol for 30 min. To ensure specific staining of nuclear DNA, cells were incubated with a staining solution containing 50 μg/mL PI and 25 μg/mL RNase A in PBS for 30 min at 37 °C, protected from light. Cell cycle phases were determined by flow cytometry using a DxFLEX flow cytometer (Beckman Coulter, Inc., Brea, CA, USA) after gating cell populations based on PE-Area versus FSC-Area plots. Data acquisition and analysis were performed using CytExpert for DxFLEX 2.0 software.

### 2.13. Colony Formation Assay

To evaluate long-term cell survival and proliferative capacity, SaOS-2 cells were seeded at a density of 1 × 10^3^ cells/well and treated with 25 nM doxorubicin in the presence or absence of terbinafine (12.5 or 25 μM) for 48 h. Following treatment, the culture medium was replaced with drug-free DMEM, and the cells were cultured for an additional 7 days. Resulting colonies were fixed with 6.0% (*v*/*v*) glutaraldehyde for 20 min and stained with 0.5% (*w*/*v*) crystal violet for 45 min. After washing three times with deionized water, colonies were imaged using the iBright™ CL-1500 system (Thermo Fisher Scientific, Inc., Waltham, MA, USA). Clusters containing more than 100 cells were defined as colonies and quantified accordingly.

### 2.14. Cell Migration and Invasion Transwell Assay

Cell migration and invasion capabilities were evaluated using a modified Boyden chamber assay. Polyvinylpyrrolidone-free polycarbonate filters (8 μm pore size; Millipore, Carrigtwohill, Ireland) were coated with 50 μL of fibronectin (10 μg/mL) for migration assays or a mixture of 50 μL fibronectin (10 μg/mL) and Matrigel (10 μg/50 μL) for invasion assays. SaOS-2 cells (1.25 × 10^5^ cells/well) were treated with various concentrations of terbinafine (0, 10, or 25 μM) and seeded into the upper chamber. The lower chamber was filled with culture medium containing 1% fetal bovine serum (FBS) as a chemoattractant. After 18 h of incubation, cells that had migrated or invaded to the lower surface of the membrane were fixed with methanol and stained with 1% (*w*/*v*) toluidine blue. The number of cells on the lower surface was subsequently counted under a microscope to quantify migratory and invasive activity.

### 2.15. Gelatin Zymography Assay

To assess gelatinolytic activity, cells were treated with terbinafine (0–25 μM) for 48 h in DMEM supplemented with 0.5% fetal bovine serum. Culture supernatants were collected, normalized for volume, and separated via 10% polyacrylamide gel electrophoresis containing 0.1% (*w*/*v*) gelatin under non-reducing conditions. Following electrophoresis, the gels were washed twice in 2.5% Triton X-100 for 30 min at room temperature to remove sodium dodecyl sulfate (SDS). Subsequently, the gels were incubated at 37 °C for 24 h in an activation buffer (50 mM Tris–HCl, 200 mM NaCl, 10 mM CaCl2, pH 7.4). The gels were stained with 0.1% (*w*/*v*) Coomassie Brilliant Blue R and destained in 30% methanol containing 10% acetic acid. Gelatinolytic activity of MMP-2 was visualized as clear bands against a blue background, and band intensity was quantified using ImageJ (v1.54r) software.

### 2.16. Western Blot Analysis

Whole-cell lysates were separated via SDS-PAGE and transferred onto a nitrocellulose membrane. The membrane was blocked and subsequently incubated with primary antibodies using a one-step solution (Bio-Helix, New Taipei, Taiwan) for 2 h at room temperature. Following primary antibody incubation, the membrane was incubated with a secondary antibody (diluted 1:10,000) in the same one-step solution for 2 h. The membrane was then washed four times with PBS containing 0.08% Tween-20 (5 min per wash) and stored in PBS prior to imaging. Protein visualization was performed using chemiluminescence, and images were captured using the iBright™ CL-1500 imaging system. Quantitative protein expression was determined by measuring band density using ImageJ (v1.54r) software.

### 2.17. Statistical Analysis

All data are presented as the mean ± standard deviation (SD) from at least three independent experiments. Statistical significance was determined using Jamovi (version 2.7.5). Differences between groups were analyzed using Student’s t-test or analysis of variance (ANOVA), as appropriate. A *p*-value of ≤ 0.05 was considered statistically significant (* *p* ≤ 0.05, ** *p* ≤ 0.005, *** *p* ≤ 0.001).

## 3. Results

### 3.1. Protein–Protein Interaction (PPI) Network Analysis and Gene Ontology (GO) Enrichment Analysis of DEGs Associated with Chemotherapy Response in Osteosarcoma

Among the 15,965 differentially expressed protein-coding genes analyzed by comparing the good and poor chemotherapy response groups across 9 osteosarcoma tissues, 819 genes met the criteria of |log_2_FC| ≥ 1.4 and *p*-value < 0.05. Among these, 446 genes were upregulated and 373 were downregulated in the poor-response group, as visualized in the volcano plot (Figure S1A and Table S1). Upon applying multiple-testing correction (|log2FC| ≥ 1.4 and p_adj_ < 0.05), 108 genes remained strictly significant, including 40 upregulated and 68 downregulated genes (Figure S2 and Supplementary Table S3). To enable robust topological network modeling and pathway-level discovery, the broader nominal DEG set (819 genes) was employed for downstream PPI network construction, MCODE cluster identification, and ClueGO functional enrichment.

To explore the protein–protein interactions (PPI) associated with chemotherapy response in osteosarcoma, a PPI network was generated using the STRING protein query feature within Cytoscape, applying a confidence threshold of 0.4 to import the upregulated differentially expressed genes (DEGs). The resulting network consisted of 427 nodes and 1132 edges (Figure S1B). Gene clusters within the PPI network were identified via the MCODE plugin and further analyzed using the ClueGO plugin to evaluate their corresponding biological functions. The top three clusters identified by MCODE (k-score = 3) were significantly enriched in distinct biological processes. Cluster 1 was associated with ribosome biogenesis and cell cycle (MCODE score = 18.05) (Figure S1C,D); Cluster 2 with ribosome biogenesis and RNA processing (MCODE score = 5.33) (Figure S1E,F); and Cluster 3 with sterol and cholesterol biosynthesis (MCODE score = 4.50) (Figure S1G,H).

**Figure 1:**
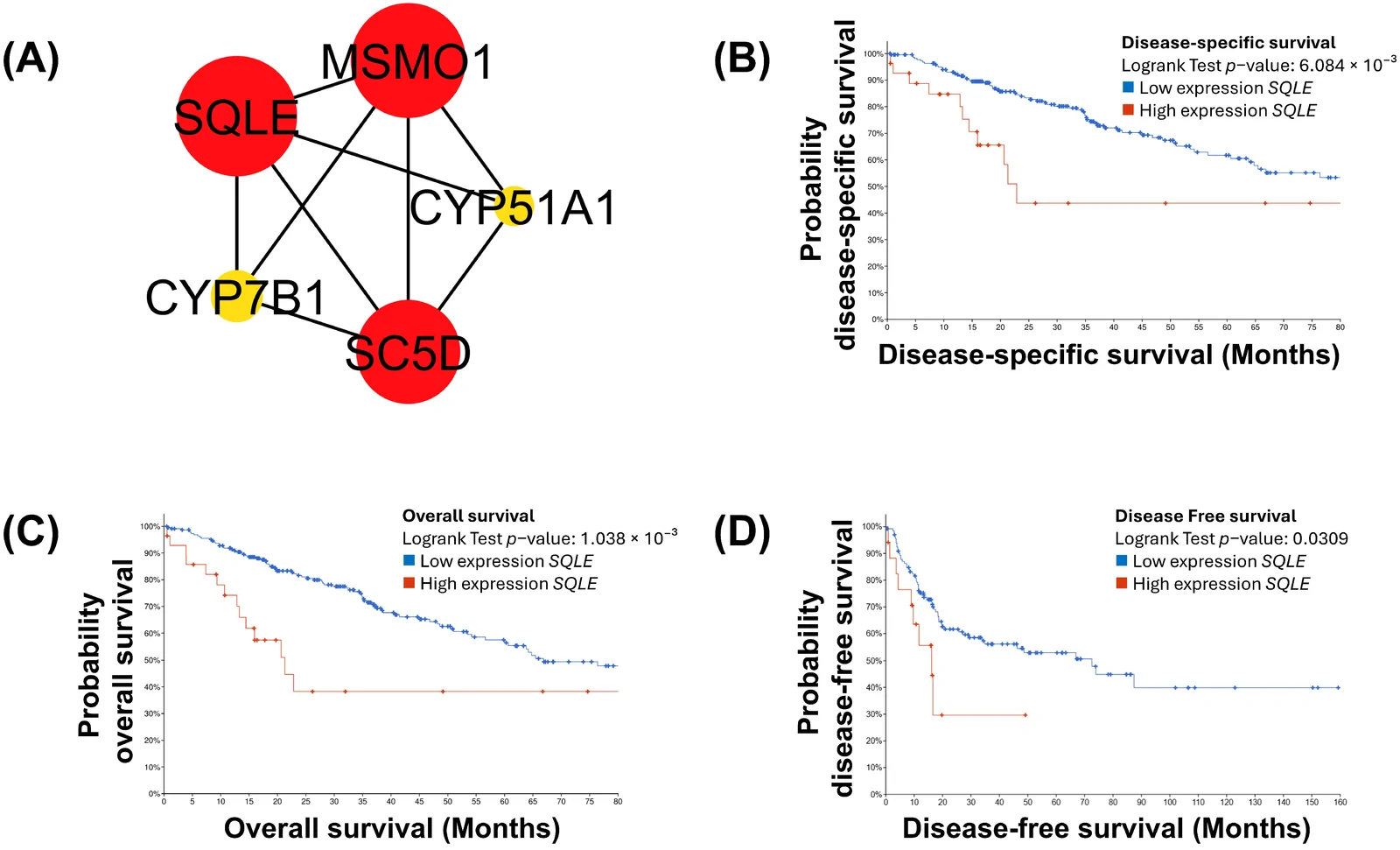
Identification of cholesterol biosynthesis hub genes and the prognostic value of SQLE in sarcoma. (**A**) Cholesterol biosynthesis hub genes analyzed using cytoHubba. (**B**) Kaplan–Meier curve showing disease-specific survival of sarcoma patients stratified by SQLE expression, analyzed via cBioPortal. (**C**) Kaplan–Meier curve showing overall survival of sarcoma patients stratified by SQLE expression, analyzed via cBioPortal. (**D**) Kaplan–Meier curve showing disease-free survival of sarcoma patients stratified by SQLE expression, analyzed via cBioPortal.

Because the cholesterol biosynthetic pathway emerged as a uniquely enriched, non-redundant biological term within ClueGO Cluster 3, we further investigated the specific molecular links between cholesterol metabolism and chemotherapy response in osteosarcoma. Consequently, the five core candidate genes within this cluster were evaluated using the cytoHubba plugin in Cytoscape to identify key functional hubs. To identify the key hub gene, the five candidates were evaluated using multiple topological analysis algorithms (including MCC, MNC, Degree, and EPC). *SQLE* (squalene epoxidase) consistently emerged as an exploratory candidate, demonstrating the highest overall topological significance and peaking specifically in the EPC metric (Figure 1A). Subsequent survival analysis using the TCGA PanCancer Atlas Sarcoma cohort revealed that elevated *SQLE* expression significantly correlated with poorer clinical outcomes, specifically shorter disease-specific (Figure 1B), overall (Figure 1C), and disease-free survival (Figure 1D). Overall, *SQLE* was nominated as an exploratory candidate target through nominal differential expression, network ranking, and biological plausibility; however, *SQLE* did not remain significant after FDR correction in this clinical transcriptomic cohort.

### 3.2. SQLE Modulates Chemotherapeutic Response in Osteosarcoma Cell Lines

**Figure 2:**
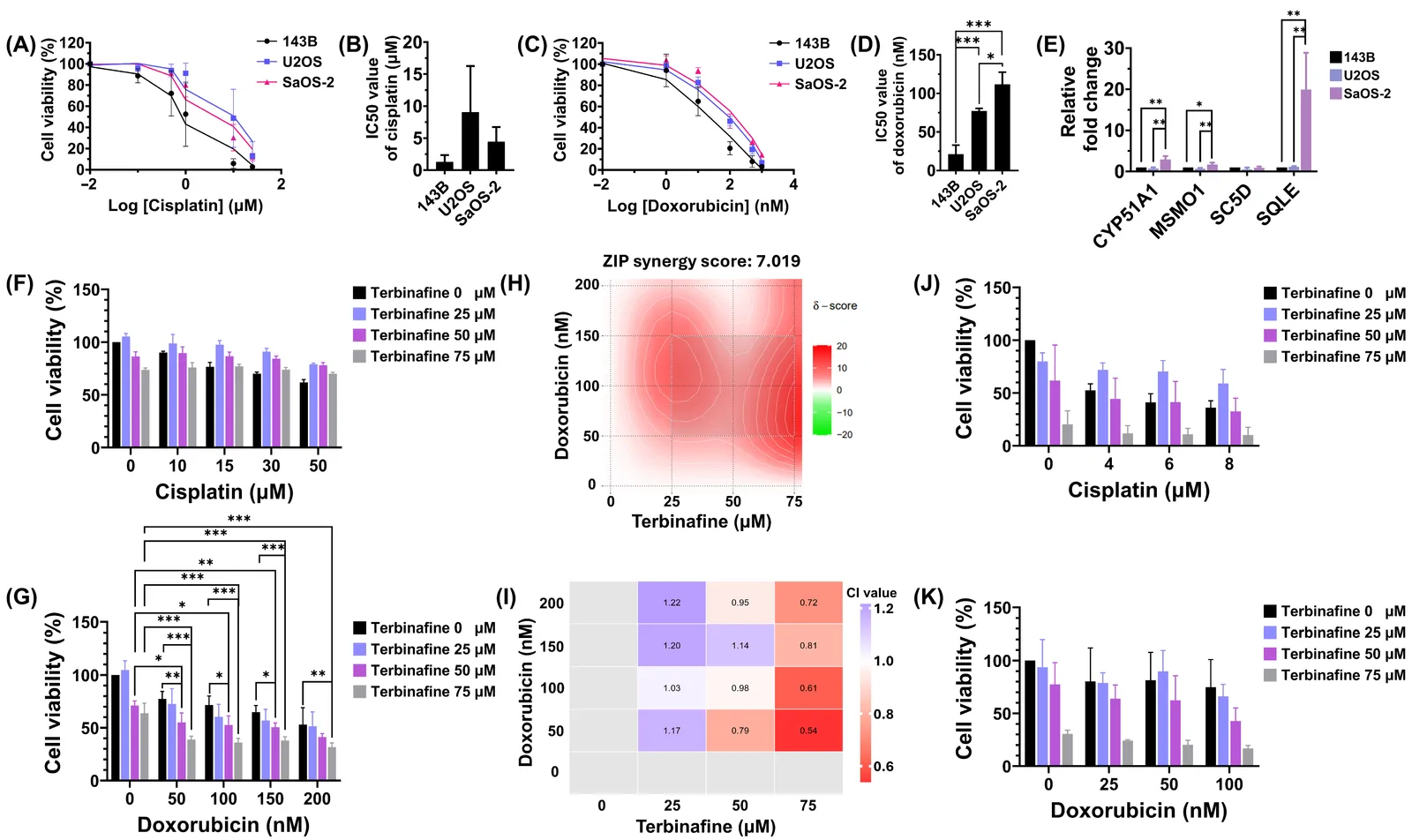
Evaluation of SQLE expression and its impact on chemotherapy response in osteosarcoma cells. (**A**) Cell viability and (**B**) IC_50_ values of osteosarcoma cell lines following cisplatin treatment for 72 h, assessed via MTT assay. (**C**) Cell viability and (**D**) IC_50_ values of osteosarcoma cell lines following doxorubicin treatment for 72 h, assessed via MTT assay. (**E**) Relative mRNA expression levels of cholesterol biosynthesis-related genes (*CYP51A1*, *MSMO1*, *SC5D*, and *SQLE*) in osteosarcoma cell lines evaluated using RT-qPCR. (**F**,**G**) Cell viability of SaOS-2 cells evaluated via MTT assay following 48 h of treatment with terbinafine (0, 25, 50, and 75 μM) in combination with various concentrations of either (**F**) cisplatin or (**G**) doxorubicin. (**H**) Contour map displaying ZIP synergy scores for the combination of doxorubicin and terbinafine in SaOS-2 cells (ZIP score > 10 indicates synergism, −10 to 10 indicates an additive effect, and <−10 indicates antagonism). (**I**) Heatmap displaying Combination Index (CI) values for the combination of doxorubicin and terbinafine in SaOS-2 cells (CI < 1 indicates synergism, CI = 1 indicates an additive effect, and CI > 1 indicates antagonism). (**J**,**K**) Cell viability of 143B cells evaluated via MTT assay following 72 h of treatment with terbinafine (0, 25, 50, and 75 μM) in combination with various concentrations of either (**J**) cisplatin or (**K**) doxorubicin. Data are expressed as the mean ± SD (*n* = 3 independent experiments). * *p* ≤ 0.05, ** *p* ≤ 0.005, and *** *p* ≤ 0.001 compared to the control group.

To investigate the role of SQLE in the chemotherapeutic response of osteosarcoma, we first determined the baseline IC_50_ values for cisplatin and doxorubicin across three distinct osteosarcoma cell lines (143B, U2OS, and SaOS-2) via MTT cell viability assays. As shown in Figure 2A,B, the IC_50_ values for cisplatin in 143B, U2OS, and SaOS-2 cells were 1.29 ± 1.04 μM, 9.07 ± 7.20 μM, and 4.46 ± 2.28 μM, respectively. Although the 143B line displayed the lowest IC_50_, these variations did not reach statistical significance. In contrast, a statistically significant difference was observed in response to doxorubicin treatment. Specifically, SaOS-2 cells demonstrated a significantly higher resistance to doxorubicin compared to both 143B and U2OS cells, with IC_50_ values at 111.87 ± 15.74 nM for SaOS-2, 77.22 ± 3.39 nM for U2OS, and 21.16 ± 11.73 nM for 143B (Figure 2C,D).

Furthermore, the baseline mRNA expression levels of the cholesterol biosynthesis-related genes, including *CYP51A1*, *MSMO1*, *SC5D*, and *SQLE*, were investigated across these three osteosarcoma cell lines via RT-qPCR. As shown in Figure 2E, the transcript levels of *CYP51A1*, *MSMO1*, and *SQLE* were significantly higher in SaOS-2 cells than in both 143B and U2OS cells. Notably, *SQLE* exhibited a striking upregulation in SaOS-2 cells, showing a 19.93 ± 8.92-fold relative increase compared to 143B, whereas U2OS cells showed only a marginal change (1.15 ± 0.16-fold relative to 143B). To evaluate the functional role of SQLE in modulating chemosensitivity, pharmacological inhibition was performed using terbinafine, a specific SQLE inhibitor, in combination with cisplatin or doxorubicin in both chemosensitive (143B) and chemoresistant (SaOS-2) cells. As shown in Figure 2F, treatment SaOS-2 cells with 50 μM cisplatin reduced cell viability to 70.12%, and co-treatment with terbinafine yielded no significant alteration in viability compared to cisplatin alone. Conversely, doxorubicin treatment at 150 nM and 200 nM reduced SaOS-2 cell viability to 64.80% and 53.05%, respectively. Notably, the addition of 75 μM terbinafine markedly enhanced the cytotoxicity, significantly reducing cell viability further to 38.01% and 31.71%, respectively (Figure 2G).

To characterize the nature of this pharmacological interaction between doxurubicin and terbinafine, the Zero Interaction Potency (ZIP) model was utilized to calculate global drug combination synergy scores. The resulting overall ZIP synergy score was 7.019, which is predominantly an additive effect (Figure 2H). To formally assess drug synergism, we calculated combination index (CI) values using the Chou-Talalay method. Interestingly, dose-specific CI analysis demonstrated that combining doxorubicin (ranging from 50 to 200 nM) with 75 μM terbinafine consistently yielded CI values less than 1 (CI < 1), confirming a true synergistic effect at these precise concentration thresholds (Figure 2I). In contrast, the combination treatments in the highly sensitive 143B cells yielded only non-significant trends toward decreased cell viability. Specifically, co-treatment with 75 μM terbinafine lowered cell viability to 10.21% when combined with 8μM cisplatin (compared to 36.19% cisplatin and 20.31% terbinafine monotherapies; Figure 2J) and to 16.81% when paired with 100 nM doxorubicin (compared to 74.81% doxorubicin and 30.56% terbinafine monotherapies; Figure 2K). None of these combined effects reached statistical significance. Taken together, these findings demonstrate that elevated baseline *SQLE* expression correlates with doxorubicin resistance in osteosarcoma cells and that pharmacological inhibition of SQLE via terbinafine selectively synergizes with doxorubicin to overcome this chemoresistance in a dose-specific manner.

### 3.3. SQLE Inhibition Synergistically Potentiates Doxorubicin-Induced SaOS-2 Cell Apoptosis

**Figure 3:**
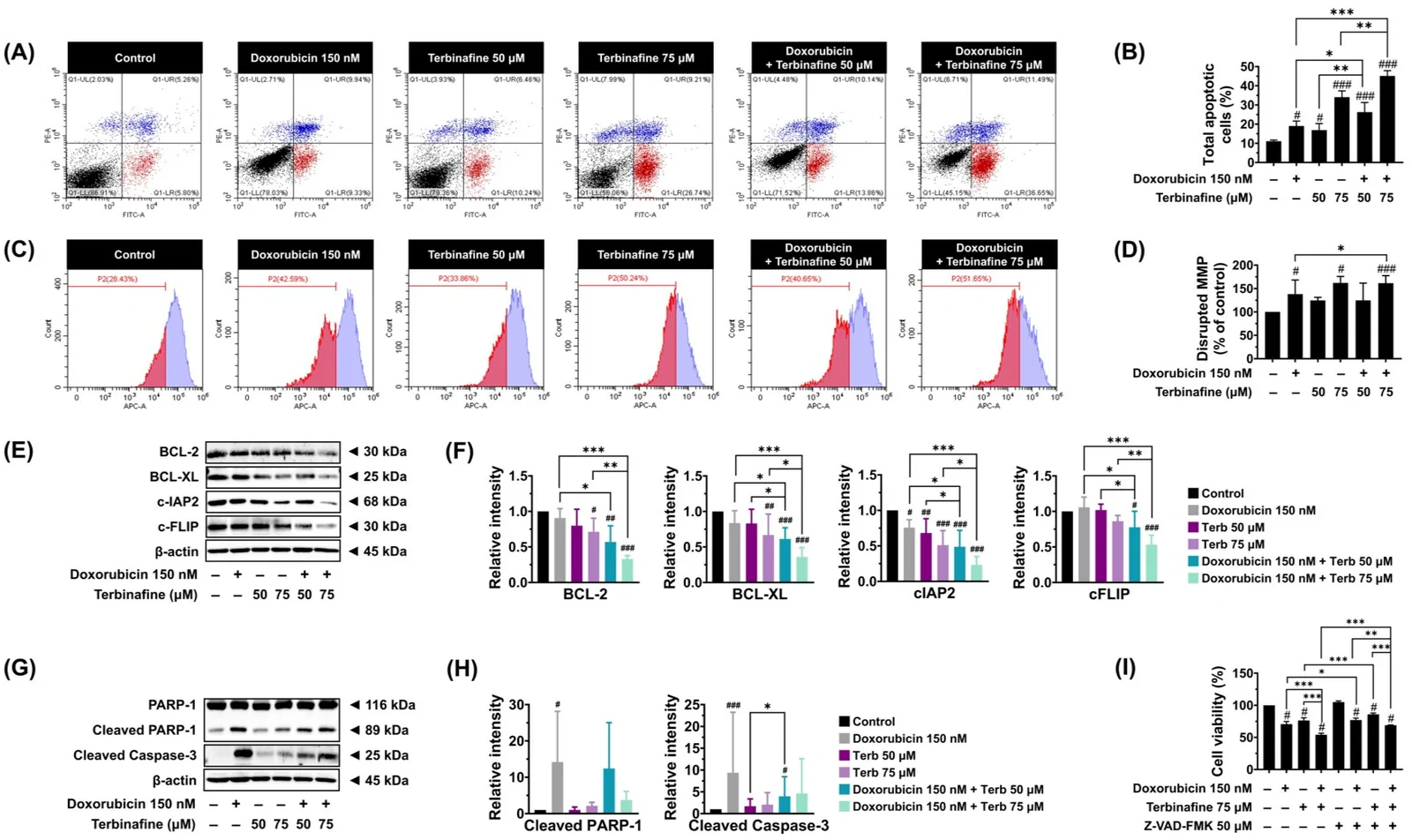
Enhancement of doxorubicin-induced SaOS-2 cell apoptosis by SQLE inhibition (**A**) Representative flow cytometry scatter plots of SaOS-2 cells following Annexin V-FITC/PI dual staining. Cells were treated with doxorubicin (150 nM), terbinafine (50 and 75 μM), or their combinations for 48 h. (**B**) Quantitative analysis showing the corresponding percentages of total apoptotic cells derived from (**A**). (**C**) Representative flow cytometry histograms of mitochondrial membrane potential (MMP) disruption in SaOS-2 cells using MitoView™ 633 staining. Cells were treated with doxorubicin (150 nM), terbinafine (50 and 75 μM), or their respective combinations for 48 h. (**D**) Quantitative analysis showing the proportion of MMP-disrupted cells normalized to the control group, derived from (**C**). (**E**,**F**) Western blot analysis of key anti-apoptotic proteins in (BCL-2, BCL-XL, c-IAP2, and c-FLIP) SaOS-2 cells treated with doxorubicin (150 nM), terbinafine (50 and 75 μM), or their respective combinations for 48 h. β-actin was used as the internal control. Representative immunoblots (**E**) and their corresponding relative band intensities calculated relative to the control group (**F**). (**G**,**H**) Western blot analysis of pro-apoptotic proteins (cleaved PARP-1 and cleaved caspase-3) in SaOS-2 cells treated with doxorubicin (150 nM), terbinafine (50 and 75 μM), or their respective combinations for 48 h. β-actin was used as the internal control. Representative immunoblots (**G**) and quantitative analysis of protein expression levels relative to the control group (**H**). (**I**) Cell viability analysis via MTT assay in SaOS-2 cells treated with 150 nM doxorubicin, 75 μM terbinafine, or their combination for 48 h, evaluated in the absence or presence of apoptosis inhibitor 50 μM Z-VAD-FMK. Data are expressed as the mean ± SD (*n* = 3 independent experiments). #, ##, and ### indicate *p* ≤ 0.05, *p* ≤ 0.005, and *p* ≤ 0.001 compared to the control group, respectively. *, **, and *** indicate *p* ≤ 0.05, *p* ≤ 0.005, and *p* ≤ 0.001 for specific comparisons between experimental groups.

To evaluate whether SQLE inhibition via terbinafine synergistically potentiates doxorubicin-induced apoptosis, SaOS-2 cells were treated with doxorubicin, terbinafine, or their combination, followed by apoptosis quantification via Annexin V/PI dual staining and flow cytometry. As shown in Figure 3A,B, treatment with doxorubicin alone at a concentration of 150 nM resulted in 18.98% apoptosis, while 50 μM and 75 μM terbinafine alone yielded 16.87% and 34.08% apoptosis, respectively. Strikingly, the combination of doxorubicin with either 50 μM or 75 μM terbinafine synergistically enhanced the apoptotic cell population to 26.26% and a robust 45.13%, respectively. Doxorubicin is widely known to induce intrinsic apoptosis by compromising mitochondrial membrane potential (MMP). To assess changes in MMP, SaOS-2 cells were stained with MitoView™ 633, a membrane-permeable dye with fluorescence intensity dependent on mitochondrial membrane potential. MMP alterations were subsequently analyzed via flow cytometry. As shown in Figure 3C,D, single-agent treatment with either 150 nM doxorubicin or 75 μM terbinafine led to a significant increase in the proportion of MMP-disrupted cells, reaching 133.60% and 161.16% of the control, respectively. Moreover, their combination further increased the population of MMP-disrupted cells compared to doxorubicin alone, exhibiting a disruption level of 166.56% of the control. These findings indicate that the disruption of MMP is a key mechanism underlying terbinafine-induced cell death. To further investigate the apoptotic machinery, the expression levels of key anti-apoptotic proteins, including BCL-2, BCL-XL, c-IAP2, and c-FLIP, were evaluated via Western blot analysis. Interestingly, the combination treatment with doxorubicin and terbinafine significantly down-regulated the expression of these anti-apoptotic proteins compared to the single-agent treatments alone (Figure 3E,F). Furthermore, pro-apoptotic markers were also assayed; as consistent with its known mechanism, doxorubicin treatment alone markedly increased the levels of cleaved PARP-1 and cleaved caspase-3 compared to the control group. Notably, however, the combination treatment did not further elevate the levels of cleaved PARP-1 and cleaved caspase-3 beyond those induced by doxorubicin alone (Figure 3G,H). To determine whether terbinafine sensitizes SaOS-2 cells to doxorubicin-induced death via apoptosis, the pan-caspase inhibitor Z-VAD-FMK was employed. As shown in Figure 3I, following co-treatment with 50 μM Z-VAD-FMK, the combination of 150 nM doxorubicin and 75 μM terbinafine still significantly decreased SaOS-2 cell viability compared to the respective single-agent treatments. Notably, the addition of Z-VAD-FMK partially rescued cell viability in both the single-agent and combination treatment groups compared to their untreated counterparts, indicating that apoptosis contributes, at least in part, to cell death. Taken together, these findings demonstrate that SQLE inhibition via terbinafine effectively potentiates doxorubicin-induced SaOS-2 cell death, a process mediated at least in part through the activation of the apoptotic pathway.

### 3.4. Terbinafine Sensitizes SaOS-2 Cells to Doxorubicin by Triggering Ferroptosis

**Figure 4:**
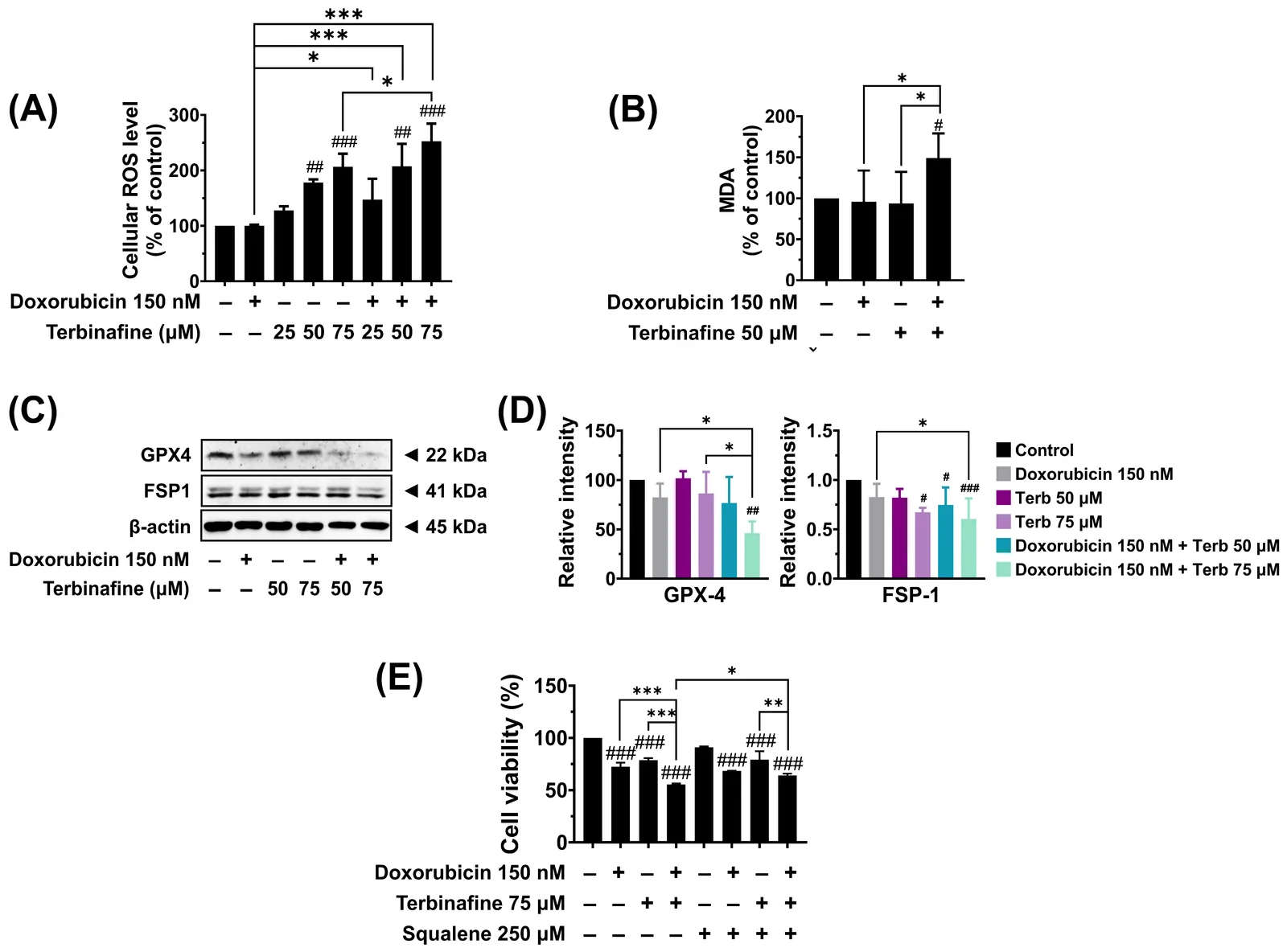
Terbinafine-mediated SQLE inhibition promotes ferroptosis to sensitize SaOS-2 cells to doxorubicin. (**A**) Intracellular reactive oxygen species (ROS) levels quantified via the DCFH-DA assay using a spectrofluorometer in SaOS-2 cells treated with 150 nM doxorubicin, terbinafine (25, 50 and 75 μM), or their respective combinations for 24 h, expressed as a percentage of the control. (**B**) Malondialdehyde (MDA) levels evaluated via the TBARS assay to assess lipid peroxidation in SaOS-2 cells treated with 150 nM doxorubicin, 50 μM terbinafine, or their combination for 1 h, expressed as a percentage of the control group. (**C**,**D**) Western blot evaluation of key ferroptosis defense proteins (FSP-1 and GPX-4) in SaOS-2 cells treated with doxorubicin (150 nM), terbinafine (50 and 75 μM), or their respective combinations for 19 h. β-actin was used as the internal control. Representative immunoblots (**C**) and quantitative analysis of protein expression levels relative to the control group (**D**). (**E**) Cell viability analysis via MTT assay in SaOS-2 cells treated with 150 nM doxorubicin, 75 μM terbinafine, or their combination for 48 h, evaluated in the absence or presence of 250 μM squalene. Data are expressed as the mean ± SD (*n* = 3 independent experiments). #, ##, and ### indicate *p* ≤ 0.05, *p* ≤ 0.005, and *p* ≤ 0.001 compared to the control group, respectively. *, **, and *** indicate *p* ≤ 0.05, *p* ≤ 0.005, and *p* ≤ 0.001 for specific comparisons between experimental groups.

Doxorubicin is a potent chemotherapeutic agent that induces cancer cell death by generating excessive ROS, which triggers DNA damage-mediated apoptosis and lipid peroxidation-driven ferroptosis. Similarly, emerging evidence shows that terbinafine suppresses tumor growth by inducing ferroptosis through iron-dependent lipid peroxidation and membrane-associated ROS accumulation. To investigate whether terbinafine induces SaOS-2 cell death via ferroptosis, we next quantified intracellular ROS and lipid peroxidation levels using DCFH-DA and TBARS assays, respectively. Additionally, the protein expression of key ferroptosis regulators, GPX-4 and FSP-1, was assessed using Western blotting. As shown in Figure 4A, intracellular ROS quantification via the DCFH-DA assay revealed that doxorubicin alone (150 nM) did not significantly elevate cellular ROS levels compared to the vehicle control. Conversely, terbinafine alone (50 μM and 75 μM) markedly induced ROS accumulation, reaching 178.12% and 206.64% of the control, respectively. Remarkably, the combination of 150 nM doxorubicin and 75 μM terbinafine synergistically elevated intracellular ROS levels to 252.42% of the control, a significantly higher level than that induced by either single-agent treatment alone. Next, the level of malondialdehyde (MDA), a major byproduct of lipid peroxidation, was evaluated via the TBARS assay to confirm the occurrence of oxidative lipid damage. As illustrated in Figure 4B, only the combination treatment consisting of 150 nM doxorubicin and 50 μM terbinafine led to a significantly higher MDA level compared to the control group. To further elucidate the molecular regulatory mechanisms governing this ferroptotic process, the expression levels of key ferroptosis defense proteins, FSP-1 and GPX-4, were examined via Western blot analysis. The results revealed that the expression level of FSP-1 was significantly decreased after co-treatment with 150 nM doxorubicin and 75 μM terbinafine compared to doxorubicin treatment alone. Moreover, this combination treatment led to an abrupt and dramatic reduction in GPX-4 levels compared to either single-agent treatment alone (Figure 4C,D). Collectively, these findings demonstrate that the combination of doxorubicin and terbinafine effectively drives SaOS-2 cell death by disrupting membrane lipid homeostasis, thereby triggering ferroptotic pathways.

Moreover, to examine whether the accumulation of squalene resulting from SQLE inhibition by terbinafine contributes to the observed drug synergism, we next evaluated the effects of exogenous squalene supplementation on cell viability. As illustrated in Figure 4E, the addition of 250 μM squalene had no significant impact on cell viability when administered alongside doxorubicin or terbinafine alone. Notably, however, the cytotoxicity of the combination treatment (150 nM doxorubicin and 75 μM terbinafine) was significantly attenuated upon squalene supplementation compared to the non-squalene condition. Furthermore, in the presence of squalene, the viability of cells subjected to the combination treatment exhibited no significant difference compared to those treated with doxorubicin alone. Thus, the addition of squalene does not mediate the synergistic cytotoxicity of SQLE inhibition and doxorubicin in SaOS-2 cells but rather rescues them from cell death. In conclusion, these findings demonstrate that the combination of doxorubicin and terbinafine enhances SaOS-2 cell sensitivity by concurrently inducing ferroptosis and partial apoptosis, a synergistic effect tightly linked to SQLE inhibition.

### 3.5. Inhibition of SQLE via Terbinafine Potentiates Doxorubicin-Mediated Proliferative Effects and Attenuates Polyploidy in SaOS-2 Cells

**Figure 5:**
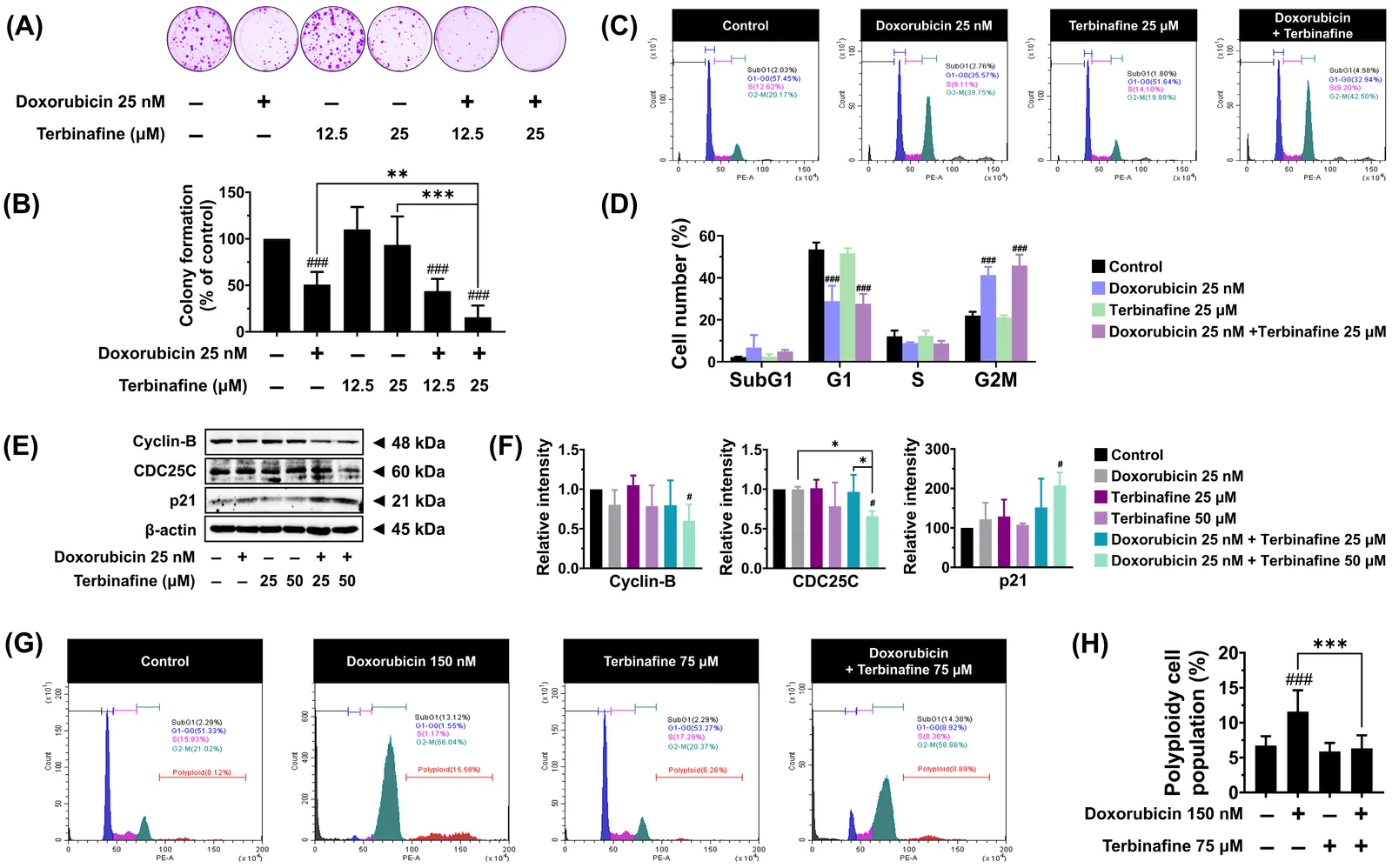
SQLE inhibition remodels the cellular response to Doxorubicin, enhancing growth suppression while preventing polyploidization in SaOS-2 cells. (**A**,**B**) Long-term cell survival evaluated via colony formation assay. (**A**) Representative images of SaOS-2 colonies after 48 h of treatment with 25 nM doxorubicin, terbinafine (12.5 and 25 μM), or their combinations, followed by drug withdrawal and a 7-day incubation in drug-free medium. (**B**) Quantitative analysis of colony formation percentage relative to the untreated control group. (**C**,**D**) Cell cycle distribution analysis evaluated via propidium iodide (PI) staining and flow cytometry. (**C**) Representative flow cytometry histograms showing SaOS-2 cell cycle profiles after 48 h of treatment with 25 nM doxorubicin, 25 μM terbinafine, or their combination. (**D**) Quantification of the percentage of cells in the SubG1, G1, S, and G2/M phases. (**E**,**F**) Expression levels of G2/M phase regulatory proteins. (**E**) Representative Western blot bands for cyclin-B, CDC25C, and p21 in SaOS-2 cells after 48 h of treatment with 25 nM doxorubicin, terbinafine (25 and 50 μM), or their combinations. β-actin was used as the internal control. (**F**) Densitometric quantification of the relative protein intensities. (**G**,**H**) Polyploid cell population analysis via propidium iodide (PI) staining and flow cytometry. Representative cell cycle histograms showing the polyploid population (**G**) and quantified percentages of polyploid cells across treatment groups (**H**). Data are expressed as the mean ± SD (*n* = 3 independent experiments). # and ### indicate *p* ≤ 0.05 and *p* ≤ 0.001 compared to the control group, respectively. *, **, and *** indicate *p* ≤ 0.05, *p* ≤ 0.005, and *p* ≤ 0.001 for specific comparisons between experimental groups.

To determine whether inhibiting SQLE via terbinafine could potentiate the doxorubicin-mediated proliferative effects, we conducted a colony formation assay to evaluate the long-term survival of SaOS-2 cells. SaOS-2 cells were treated with 25 nM doxorubicin, terbinafine (12.5 and 25 μM), or their combinations for 48 h, followed by drug withdrawal and a 7-day incubation in drug-free medium. Treatment with doxorubicin alone decreased the colony formation percentage to 50.86% relative to the control group. Moreover, the results demonstrated that the combination of 25 nM doxorubicin and 25 μM terbinafine significantly suppressed colony-forming ability compared to both the control and single-agent treatment groups, dropping to 15.67% of the control, representing a 3.25-fold reduction compared to doxorubicin alone (Figure 5A,B). Furthermore, cell cycle distribution was evaluated via propidium iodide (PI) staining and flow cytometry. Doxorubicin alone and the combination treatment significantly induced cell cycle arrest at the G2/M phase compared to the control group (Figure 5C,D). To investigate the molecular basis of the $G_{2}/M$ phase arrest, we examined the expression of key checkpoint regulators (cyclin-B, CDC25C, and p21) using Western blotting. The combination of 25 nM doxorubicin and 50 μM terbinafine significantly decreased cyclin-B expression compared to the control group. Notably, this combination treatment also markedly downregulated CDC25C expression compared to both the control and single-agent groups (Figure 5E,F). Moreover, the combination of 25 nM doxorubicin and 50 μM terbinafine significantly increased p21 protein expression level when compared to the control group. Taken together, these findings indicate that the combination treatment effectively inhibits cell proliferation by inducing G2/M phase cell cycle arrest.

Polyploidy in cancer is defined as the presence of more than two complete sets of chromosomes, acting as a hallmark of tumor progression. These cells are frequently induced by genotoxic drugs such as cisplatin and doxorubicin. Once formed, they promote tumor survival, accelerate metastasis, and drive disease relapses and drug resistance. Therefore, we investigated whether targeting SQLE via terbinafine could attenuate the development of doxorubicin-induced polyploid populations in SaOS-2 cells by using PI staining. The results revealed that treatment with 150 nM doxorubicin alone dramatically increased the percentage of polyploid cells to 11.59%, representing a 1.73-fold change relative to the control group. Interestingly, upon co-treatment with 75 μM terbinafine, the percentage of polyploid cells significantly decreased back to the baseline control level compared to doxorubicin treatment alone (Figure 5G,H). This result indicates that a combination strategy successfully suppresses the emergence of therapy-induced polyploid populations, offering a promising approach to prevent drug resistance and disease relapse in osteosarcoma.

### 3.6. Terbinafine Suppresses SaOS-2 Cell Migration and Invasion

**Figure 6:**
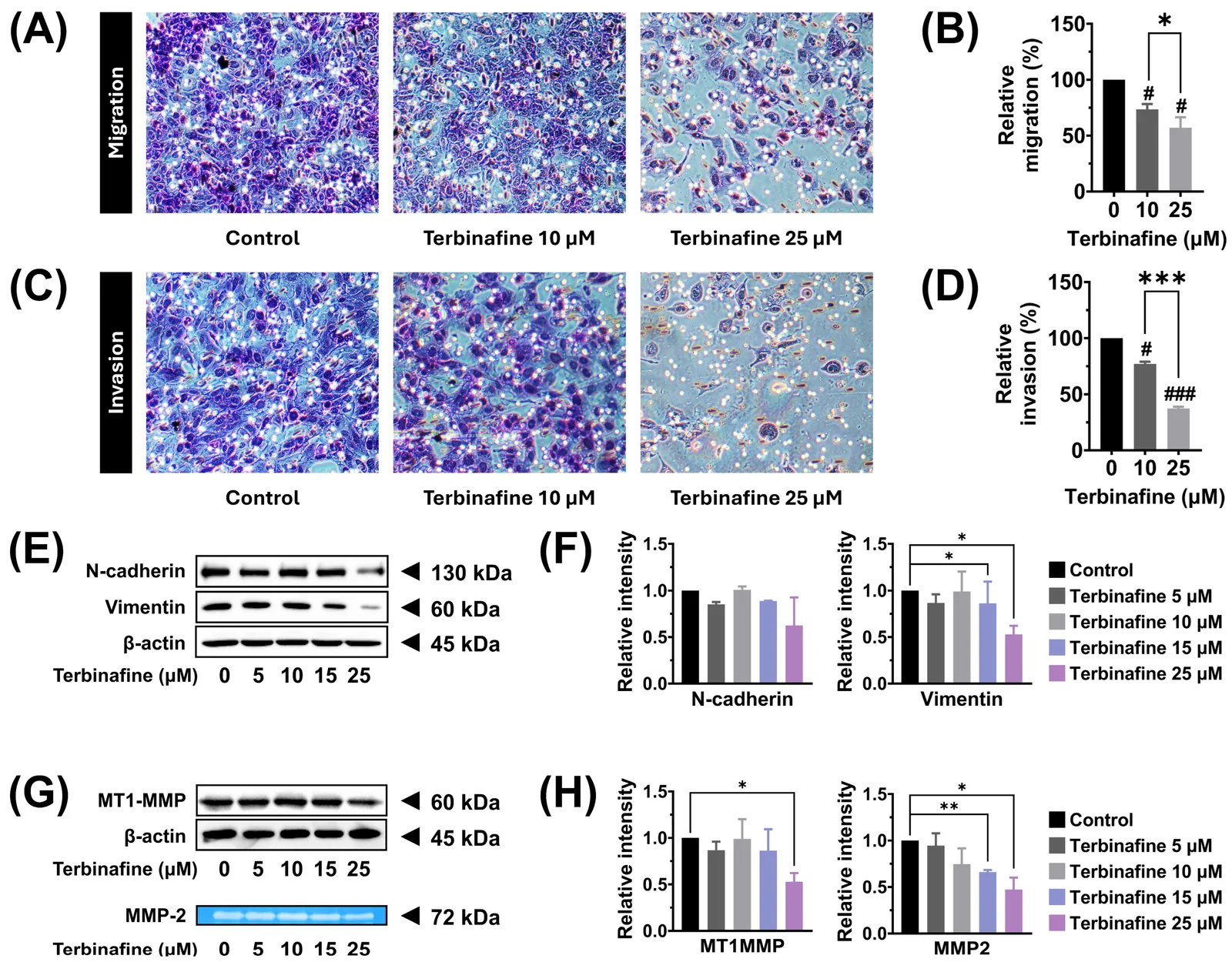
Inhibitory effects of terbinafine on SaOS-2 cell migration and invasion. (**A**,**B**) Transwell cell migration assay. (**A**) Representative microscopic images of migrated SaOS-2 cells treated with non-cytotoxic concentrations of terbinafine (10 and 25 μM) for 18 h (10× magnification). (**B**) Quantitative analysis of the relative percentage of migrated cells compared to the untreated control group. (**C**,**D**) Transwell cell invasion assay. (**C**) Representative microscopic images of invaded SaOS-2 cells passing through Matrigel-coated membranes after 18 h treatment with terbinafine (10 and 25 μM) (10× magnification). (**D**) Quantitative analysis of the relative percentage of invasive cells compared to the untreated control group. (**E**,**F**) Expression levels of epithelial–mesenchymal transition (EMT) protein markers. (**E**) Representative Western blot bands for N-cadherin and Vimentin in SaOS-2 cells after treatment with various concentrations of terbinafine (5, 10, 15, and 25 μM) for 48 h. β-actin was used as the internal control. (**F**) Densitometric quantification of the relative protein intensities. (**G**,**H**) Expression and enzymatic activity of extracellular matrix (ECM) degradation enzymes. (**G**) Representative Western blot bands for MT1MMP and a representative gelatin zymography gel showing the enzymatic activity of MMP2 in SaOS-2 cells treated with terbinafine (5, 10, 15, and 25 μM) for 48 h. β-actin was used as the internal control. (**H**) Densitometric quantification of relative MT1MMP protein expression levels and MMP2 gelatinolytic activity. Data are expressed as the mean ± SD (*n* = 3 independent experiments). #, and ### indicate *p* ≤ 0.05 and *p* ≤ 0.001 compared to the control group, respectively. *, **, and *** indicate *p* ≤ 0.05, *p* ≤ 0.005, and *p* ≤ 0.001 for specific comparisons between experimental groups.

To investigate the role of SQLE in the migration and invasion of SaOS-2 cells, the cells were treated with terbinafine and analyzed using Transwell assays. As shown in Figure 6A,B, terbinafine treatment at 10 and 25 μM significantly suppressed SaOS-2 cell migration to 73.47% and 57.11% of the control, respectively. Similarly, terbinafine demonstrated a significant dose-dependent inhibition of cell invasion; 10 μM terbinafine reduced invasions to 77.09%, while 25 μM markedly decreased invasive capacity to 37.34% compared to the control group (Figure 6C,D). To elucidate the underlying molecular mechanism, epithelial–mesenchymal transition (EMT) markers were evaluated via Western blot analysis. Treatment with 25 μM terbinafine showed a downward trend in N-cadherin expression, whereas 15 and 25 μM terbinafine significantly downregulated vimentin protein levels (Figure 6E,F). Furthermore, the expression and activity of extracellular matrix (ECM) degradation enzymes were evaluated. Western blot analysis revealed that 25 μM terbinafine significantly decreased MT1-MMP levels relative to the control. Concurrently, gelatin zymography demonstrated that 15 and 25 μM terbinafine significantly diminished MMP-2 enzymatic activity compared to the control group (Figure 6G,H). Collectively, these findings indicate that SQLE inhibition via terbinafine effectively suppresses the migration and invasion of SaOS-2 cells via downregulation of EMT markers and ECM-degrading enzymes.

### 3.7. Effect of Terbinafine on the PI3K/AKT/mTOR and MAPK Signaling Cascades in SaOS-2 Cells

**Figure 7:**
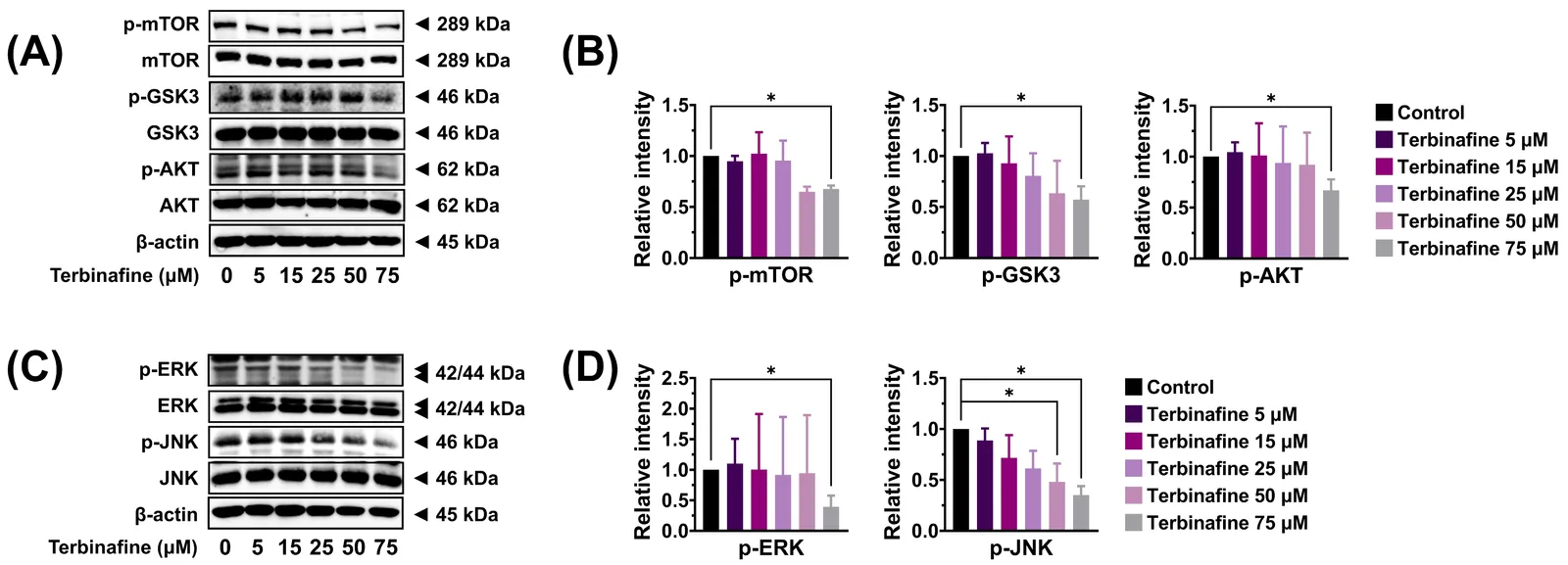
Inhibitory effects of terbinafine on the PI3K/AKT/mTOR and MAPK pathways in SaOS-2 cells. (**A**) Western blot analysis of key components in the PI3K/AKT/mTOR pathway, including p-mTOR, mTOR, p-GSK3, GSK3, p-AKT, and AKT, in SaOS-2 cells treated with various concentrations of terbinafine (0, 5, 15, 25, 50, and 75 μM) for 24 h. β-actin was used as the internal control. (**B**) Quantitative densitometric analysis of the relative protein intensities of p-mTOR, p-GSK3, and p-AKT normalized to their respective total proteins or loading control. (**C**) Western blot analysis of key members in the MAPK signaling cascade, including p-ERK, ERK, p-JNK, and JNK, following terbinafine treatment under the same conditions. β-actin was used as the internal control. (**D**) Quantitative densitometric analysis of the relative protein intensities of p-ERK and p-JNK. Data are expressed as the mean ± SD (*n* = 3 independent experiments). * indicates *p* ≤ 0.05 compared with the control group.

Given that SQLE has been reported to activate multiple oncogenic pathways, including the PI3K/AKT/mTOR and ERK signaling cascades, which regulate the expression of survival and invasive proteins, we investigated the effects of terbinafine on the activation of the PI3K/AKT/mTOR and MAPK signaling pathways by determining the phosphorylation with Western blot analysis. These pathways were comprehensively investigated via Western blot analysis to elucidate the molecular mechanisms underlying SQLE inhibition via terbinafine in SaOS-2 cells. As shown in Figure 7A,B, terbinafine treatment for 24 h, particularly at 75 μM, significantly decreased the phosphorylation levels of key members in the PI3K/AKT/mTOR pathway, including p-mTOR, p-GSK3, and p-AKT, which serve as critical components of the signaling network governing cell growth and survival. Moreover, two key pathways of the MAPK (Mitogen-Activated Protein Kinase) cascade, namely ERK and JNK, were evaluated (Figure 7C,D). Regarding the p-ERK pathway, which is well-known to drive oncogenic progression and cell migration, terbinafine treatment at 75 μM resulted in a significant reduction in p-ERK levels, consistent with the suppressed cell motility observed in Figure 7C,D. In addition, the alterations in p-JNK were examined, as this pathway is widely documented to modulate stress responses and cell fate. The results demonstrated that terbinafine treatment for 24 h, especially at a concentration of 75 μM, significantly decreased the phosphorylation levels of JNK (Figure 7C,D). Therefore, the disruption of these crucial survival and mitogenic pathways suggests that these molecular inhibitory effects functionally translate into the suppression of oncogenic cellular phenotypes essential for osteosarcoma progression.

## 4. Discussion

Innate chemotherapy resistance remains a significant clinical challenge in osteosarcoma management, as it inherently diminishes treatment efficacy and often contributes to poor clinical outcomes. As chemoresistance acts as a primary barrier to successful cancer management, it serves as a major driver for both tumor recurrence and metastatic progression [13]. Driven by these challenges, this study aims to elucidate the molecular mechanisms underlying chemosensitivity in high-grade osteosarcoma through an integrated in silico and in vitro investigation, utilizing transcriptomic analysis followed by experimental validation. In this study, transcriptomic analysis nominated *SQLE* as an exploratory candidate hub gene associated with poor chemotherapy response. Because these data were derived from chemo-naïve biopsies, these exploratory findings support further investigation into whether elevated *SQLE* expression serves as a potential metabolic feature linked to intrinsic drug resistance and metastatic potential in osteosarcoma.

SQLE is the second rate-limiting enzyme in cholesterol biosynthesis, catalyzing the oxygenation of squalene to 2,3(S)-monooxidosqualene [14]. It is frequently overexpressed in various cancers, including breast [15], prostate [16], pancreatic [17], ovarian [18], and bladder [19]. High *SQLE* levels act as oncogenes by promoting tumor growth, metastasis, and therapy resistance. Previous studies in pancreatic cancer demonstrate profound *SQLE* upregulation that strongly correlates with poor clinical outcomes. In this context, SQLE promotes progression by driving de novo cholesterol biosynthesis, which stabilizes lipid rafts and hyperactivates the Src/PI3K/AKT survival axis. Concurrently, blocking this enzyme induces lethal endoplasmic reticulum stress via squalene accumulation [17]. Conversely, genetic silence of *SQLE* in pancreatic cancer cells causes an accumulation of cellular squalene, leading to the suppression of mitochondrial biogenesis and metabolism, which ultimately impairs tumor growth [20]. Moreover, elevated *SQLE* expression in ovarian cancer promotes tumorigenesis by protecting malignant cells from iron-dependent ferroptosis [18]. On the other hand, SQLE drives bladder cancer cell growth by boosting mitochondrial oxidative phosphorylation [19]. Crucially, in the context of osteosarcoma, high *SQLE* expression has been associated with significantly shorter overall survival. Furthermore, loss- and gain-of-function experiments have indicated that silencing *SQLE* suppresses osteosarcoma cell proliferation, migration, and invasion, whereas its overexpression exerts the opposite effects by activating the TGF-β/SMAD signaling pathway [21]. Bioinformatic analysis of our transcriptomic data indicated that a cluster of candidate genes within the cholesterol biosynthetic pathway was associated with poor chemotherapeutic response. Specifically, the downstream cholesterol biosynthesis enzymes *SC5D* and *MSMO1* were identified as candidate hub genes that remained statistically significant following FDR correction. In contrast, *SQLE* met the nominal expression threshold (*p* < 0.05) but did not remain significant after FDR adjustment. Nevertheless, clinical survival analysis using the TCGA PanCancer Atlas Sarcoma cohort revealed that elevated *SQLE* expression significantly correlated with poorer clinical outcomes, specifically shorter overall, disease-specific, and disease-free survival. Because a formal FDR-corrected pathway-enrichment analysis was not executed across the entire transcriptomic dataset, these transcriptomic findings support continued investigation into the functional role of cholesterol metabolism in osteosarcoma chemoresistance, rather than establishing definitive confirmation of the entire pathway.

To validate these transcriptomic insights in vitro, the functional role of SQLE was evaluated in SaOS-2 cells. Intriguingly, our combinational assays revealed a distinct, drug-specific interaction; while terbinafine treatment failed to sensitize SaOS-2 cells to cisplatin, it exhibited a profound synergistic cytotoxicity when combined with doxorubicin. This contrasting efficacy is likely governed by the distinct modes of action characterizing these two chemotherapeutic agents. Cisplatin primarily exerts its cytotoxic effects within the nucleus by inducing DNA cross-links [22], a process independent of lipid homeostasis. Conversely, beyond inhibiting topoisomerase II, doxorubicin is converted into an unstable semiquinone radical that reacts with oxygen to continuously generate superoxide anions and hydrogen peroxide [23], ultimately triggering catastrophic lipid peroxidation [24]. Cellular SQLE serves as a key rate-limiting enzyme in cholesterol biosynthesis and a critical regulator of oxidative homeostasis [25]. Inhibiting SQLE triggers a profound accumulation of squalene. This lipid congestion within the endoplasmic reticulum induces severe ER stress and drives an uncontrolled spike in cellular ROS [17,18]. Therefore, the combination of terbinafine and doxorubicin exacerbates intracellular oxidative stress, thereby synergistically inducing SaOS-2 cell death.

Beyond general cytotoxicity, we further investigated the precise mode of cell death elicited by this combination therapy. Flow cytometric analysis using Annexin V/PI staining initially indicated a significant increase in the total apoptotic cell population upon doxorubicin and terbinafine co-treatment. Interestingly, the expression of downstream apoptotic executioners, cleaved caspase-3 and cleaved PARP-1, in combination treatment of doxorubicin and terbinafine did not show a corresponding significant increase compared to that induced by doxorubicin alone, despite a marked decrease in anti-apoptotic proteins such as Bcl-2, Bcl-xL, cIAP2, and cFLIP. This discrepancy strongly suggests that classical apoptosis may not serve as the primary mechanism of the combination treatment. This phenomenon can be explained by the physical alterations of the cell membrane during specific cell death pathways such as ferroptosis. The overproduction of ROS induced oxidative damage to the plasma membrane and created nano-size gaps. These membrane defects facilitate the premature influx of membrane-impermeable dyes like PI during the early stages of cell death, which later culminates in dual-positive staining. Because conventional Annexin V/PI assays cannot distinctively separate this staining profile from late apoptosis [26], the combination treatment of doxorubicin and terbinafine markedly increases the population of PI-stained cells through this distinct mechanism. To definitively delineate these overlapping phenotypes, the pan-caspase inhibitor Z-VAD-FMK was utilized. Although the addition of Z-VAD-FMK partially rescued cell viability, the combination treatment still sustained a significantly higher cytotoxic rate compared to the single-agent controls. Taken together, these findings confirm that while apoptosis is partially involved, it is not the sole execution pathway. Supporting this notion, emerging evidence in other malignancies, such as ovarian cancer, has demonstrated that SQLE depletion triggers ferroptosis, whereas its overexpression confers resistance against this specific cell death pathway [18].

Driven by these insights, we evaluated whether the terbinafine-doxorubicin synergy operates via ferroptosis, an iron-dependent form of regulated cell death characterized by overwhelming lipid peroxidation and membrane-associated ROS accumulation. Notably, the combination treatment effectively suppressed the cellular anti-ferroptosis machinery, as demonstrated by the marked downregulation of FSP-1 and GPX-4 expressions. This suppression coincided with a profound elevation in intracellular ROS levels exceeding those of the single-agent controls. Furthermore, the co-treatment was uniquely capable of inducing the accumulation of MDA, a definitive end-product of lipid peroxidation. Taken together, these data establish that SQLE inhibition by terbinafine sensitizes SaOS-2 cells to doxorubicin by activating concurrent, parallel pathways of apoptosis and ferroptosis. To determine whether this catastrophic membrane lipid stress and subsequent cell death are specifically linked to the SQLE-regulated metabolic axis, we utilized exogenous squalene supplementation. Strikingly, while external squalene supplementation exerted no observable impact on the cell viability of the single-agent groups, it selectively and profoundly rescued SaOS-2 cells from the terbinafine-doxorubicin synergistic lethality. Under squalene-enriched conditions, the cell viability of the combination group exhibited a dramatic surge, completely neutralizing the synergistic effect of terbinafine and returning to a level that was statistically non-significant compared to doxorubicin treatment alone. This finding contrasts with previous reports indicating that the intracellular accumulation of squalene within the endoplasmic reticulum and mitochondria, which is triggered by SQLE inhibition, induces cancer cell death [17]. Given that squalene acts as an endogenous antioxidant [27], it is highly probable that this rescue effect is driven by its free-radical scavenging properties, which mitigate the oxidative stress induced by terbinafine alone or in combination with doxorubicin. This hypothesis is supported by our observation that exogenous squalene supplementation successfully rescued cell viability in both the terbinafine single-agent and combination treatment groups. Collectively, these findings demonstrate that the synergistic cytotoxicity of doxorubicin and terbinafine is fundamentally predicated on oxidative damage of cellular lipid, which overpowers the cellular defense machinery to drive cell death.

Next, the impact of SQLE inhibition on SaOS-2 cell proliferation and cell cycle dynamics was investigated. Proportions of cells in distinct cell cycle phases revealed that while terbinafine monotherapy did not alter cell cycle distribution compared to the untreated control, both doxorubicin monotherapy and the combination treatment triggered G2/M phase cell cycle arrest. To molecularly confirm this G2/M blockade, we investigated specific mitotic protein markers, including cyclin-B, CDC25C, and p21. The combination treatment significantly decreased the expression of cyclin-B and CDC25C; this concomitant downregulation suppresses M-CDK complex activation, thereby molecularly impeding entry into mitosis and enforcing the G2/M phase arrest. Concurrently, the combination treatment hyper-induced the expression of the cyclin-dependent kinase inhibitor p21, which directly binds to and inactivates the CDK1/cyclin-B complex, cooperatively tightening the G2/M checkpoint block. Intriguingly, although both doxorubicin monotherapy and the combination regimen obstruct cell proliferation within the G2/M phase to a similar percentage, they dictate fundamentally distinct cell fates during this mitotic disaster. The addition of terbinafine drives a massive increase in lipid peroxidation and subsequent membrane permeabilization, thereby chemosensitizing the cells. Consequently, the colony-forming ability and long-term reproductive survival of SaOS-2 cells are drastically diminished exclusively under the combination treatment. As expected, doxorubicin monotherapy demonstrated intrinsic anti-proliferative activity, significantly reducing colony formation to approximately half of the control levels. This substantial reduction validates the established cytotoxic potency of doxorubicin as a standard chemotherapeutic agent. However, this single-agent exposure leaves behind a prominent subpopulation of viable, surviving cells capable of establishing long-term colonies, which clinically represents a major reservoir for subsequent drug resistance and disease relapse. Crucially, the addition of terbinafine delivered a devastating synergistic blow to this remaining fraction, driving the clonogenic potential down to negligible levels that were significantly lower than both the untreated control and doxorubicin monotherapy. This profound suppression underscores that while terbinafine alone possesses no inherent anti-clonogenic effect, its cooperative interaction with doxorubicin effectively eliminates the survival window of the doxorubicin-resistant fraction, transforming a partially effective cytostatic treatment into a comprehensively lethal therapeutic strategy.

Crucially, cell cycle analysis via PI staining revealed a significant accumulation of a polyploid cell population following doxorubicin monotherapy. This finding strongly aligns with previous literature establishing that sublethal doxorubicin exposure drives a subpopulation of cancer cells into temporary endopolyploidy, a state characterized by repeated rounds of DNA replication without cell division. This polyploid transition serves as a critical adaptive survival mechanism, ultimately facilitating chemoresistance and driving long-term cancer recurrence [28,29]. Doxorubicin induces polyploidy in cancer cells primarily as a response to DNA damage stress [29], forcing cells out of normal division and into a process called endoreplication or mitotic slippage [30]. Moreover, increased ROS accumulation triggers an oxidative stress response that leads to polyploidization [29,31]. Instead of undergoing immediate death, these cells utilize polyploidization as a survival mechanism to cope with genomic instability. Recent evidence highlights that polyploid cells possess a remarkably higher tolerance to substantial DNA damage, allowing them to mitigate its cytotoxic impact and delay cell cycle arrest to establish protective dormancy rather than undergoing apoptosis [32]. Our study also demonstrated that doxorubicin induced the population number of polypoid cells. Interestingly, the addition of terbinafine completely abolished this doxorubicin-induced polyploid population, returning the polyploidy rate back to control levels. This drastic decline indicates that the terbinafine-mediated SQLE blockade effectively closes this survival window. By triggering an overwhelming surge of intracellular ROS while simultaneously dismantling antioxidant defenses via FSP-1 and GPX-4 downregulation, this combination treatment compromises lipid bilayer integrity. This vulnerability strips potential polyploid giant cancer cell precursors of their intrinsic stress tolerance, forcing them to apoptotic and ferroptotic pathways before they can stabilize their genomic reservoir. Consequently, the terbinafine–doxorubicin regimen targeted abolishes this adaptive escape route, highlighting its significant therapeutic promise in preventing long-term chemoresistance and recurrence in osteosarcoma.

Consistent with previous oncological reports, SQLE has been widely characterized as a poor prognostic biomarker intricately linked to aggressive cancer behaviors and metastatic progression across various malignancies [33,34,35]. For instance, genetic knockdown of *SQLE* has been demonstrated to significantly impair the proliferation, migration, and invasive capabilities of cervical cancer cells, while its overexpression strongly correlates with lymphovascular invasion and pathologic TNM stage through the driving of EMT [33]. In alignment with these observations, our functional characterization utilizing transwell migration and invasion assays clearly demonstrated that pharmacological inhibition of SQLE via terbinafine significantly reduced the number of migrated and invasive SaOS-2 osteosarcoma cells in a concentration-dependent manner. To unveil the underlying molecular machinery behind this anti-metastatic effect, we further evaluated key protein markers governing cellular motility and extracellular matrix remodeling. Interestingly, the secretion level of MMP-2 demonstrated acute sensitivity to terbinafine, showing a statistically significant reduction starting at a lower concentration. Given that MMP-2 is a key enzyme for degrading the extracellular matrix, its early-phase suppression directly accounts for the significant functional decline in SaOS-2 invasion observed at the same dose. However, a high concentration of terbinafine markedly downregulated the expression of vimentin, a crucial mesenchymal intermediate filament marker, suggesting a potential reversion or halting of the EMT program, while concurrently abolishing the expression of MT1-MMP. Taken together, these findings verify that targeting the SQLE axis with non-toxic doses of terbinafine molecularly disrupts the migratory and matrix-degrading apparatus of osteosarcoma cells, highlighting its profound efficacy in restricting the metastatic phenotype.

To deeply understand the intracellular signaling networks through which SQLE inhibition drives its anti-cancer effects, we extensively profiled the primary survival and mitogenic cascades in SaOS-2 cells. Our Western blot investigations revealed that terbinafine treatment leads to a coordinated suppression of multiple key pathways, prominently achieving a significant downregulation at the higher concentration of 75 μM. Specifically, the marked decrease in the phosphorylation of AKT, mTOR, and GSK3 highlights a major collapse in the PI3K/AKT/mTOR signaling axis. Given that this axis is universally recognized as a central engine driving osteosarcoma cell survival, metabolic reprogramming, proliferation, and metastasis [36,37,38], its suppression after terbinafine treatment is highly consistent with our Western blot findings showing the downstream downregulation of key metastatic and mesenchymal markers (vimentin, MT1-MMP, and MMP-2). Consequently, this coordinated inhibition directly accounts for the observed impairment of migratory and invasive behaviors in SaOS-2 cells. Concurrently, the significant reduction in p-ERK levels further clarifies the molecular basis behind the weakened cell motility found in our transwell assays. Given that the ERK signaling cascade is a well-established driver of the epithelial-to-mesenchymal transition (EMT) [37], this suppression likely contributes to the observed downregulation of mesenchymal markers such as vimentin and MT1-MMP, thereby confirming that SQLE blockade effectively cuts off the mitogenic signals required for active cell mobility.

Beyond these well-established survival cascades, the most notable and intriguing finding from our molecular profiling is the profound, concentration-dependent decrease in p-JNK levels following terbinafine treatment. While c-Jun N-terminal kinase (JNK) signaling is notorious for exhibiting highly complex, dual roles as either a tumor promoter or tumor suppressor depending on the cellular context and tumor microenvironment [39]. Emerging evidence highlights JNK as a master protein kinase governing osteosarcoma cell fate, where its chemical inhibition has been shown to strongly attenuate tumor proliferation and metastatic progression both in vitro and in vivo [40]. Therefore, the robust suppression of p-JNK observed in our study serves as a critical mechanism explaining the loss of aggressive oncogenic phenotypes in SaOS-2 cells, as evidenced by the concomitant downstream downregulation of vimentin, MT1-MMP, and MMP-2. Furthermore, the recent literature indicates a tight regulatory link between the JNK pathway and lipid homeostasis, noting that JNK dynamics are intimately modulated by reactive oxygen species, lipid peroxide accumulation, and glutathione status, which collectively determine cancer cell vulnerability to lipid-driven cell death such as ferroptosis [41]. Because terbinafine specifically disrupts upstream lipid biosynthesis via SQLE inhibition, this striking drop in p-JNK strongly implies that the drug induces an acute crisis in cellular lipid order and stress-response machinery, ultimately shifting the cell fate away from survival.

Taken together, these molecular insights verify that the anti-osteosarcoma efficacy of terbinafine is not mediated by a single isolated event but rather through a comprehensive multi-pathway blockade. By simultaneously disrupting the PI3K/AKT/mTOR survival core, suppressing the ERK migratory signal, and inhibiting the lipid-sensitive JNK stress axis, SQLE targeting effectively shuts down the essential signaling machinery required for osteosarcoma progression. These findings strongly highlight the therapeutic potential of targeting SQLE as a versatile strategy to overcome the aggressive features of bone malignancies.

Several limitations of this study should be acknowledged. First, the clinical transcriptomic analysis was conducted on a relatively small cohort (*n* = 9). Second, while the utilization of treatment-naïve biopsy tissues represents a key strength for identifying intrinsic, baseline molecular drivers before therapeutic exposure, patient categorization was strictly based on post-chemotherapy histological response (% tumor necrosis). Consequently, all poor responders in this cohort also exhibited subsequent metastatic progression during clinical follow-up, reflecting an intrinsic high-risk, aggressive phenotype tightly coupled with dismal clinical outcomes that overlap with mechanisms of intrinsic chemoresistance. Third, applying stringent FDR corrections naturally reduced the number of statistically significant genes. Consequently, *SQLE* is appropriately framed as an exploratory candidate target, selected through network clustering and biological pathway relevance, rather than a definitively established clinical biomarker. Furthermore, while previous studies have reported the single-agent in vivo anticancer efficacy of terbinafine in other malignancies such as colorectal and oral cancers [42,43,44], its synergistic potential when combined with standard chemotherapeutics (such as doxorubicin or cisplatin) remains entirely unexplored in in vivo models across all cancer types. Although our in vitro functional assays provide the first proof-of-concept that SQLE inhibition via terbinafine sensitizes osteosarcoma cells to doxorubicin, we acknowledge the absence of in vivo animal validation as a study limitation. Furthermore, according to the World Health Organization (WHO) classification, osteosarcoma encompasses diverse histopathological subtypes with distinct clinical behaviors and tumor microenvironments (TME), broadly categorized into central/medullary and surface/peripheral forms [45]. In this study, our functional assays were established using SaOS-2 cells, a well-characterized model representing high-grade osteoblastic central osteosarcoma, the predominant conventional subtype accounting for 80% of all clinical cases [45]. However, variations in extracellular matrix composition, vascularization, and immune/stromal cell heterogeneity across other osteosarcoma subtypes and their respective TMEs could influence SQLE-mediated cholesterol reliance and drug susceptibility. Future prospective studies with larger, histologically validated clinical cohorts, diverse subtype-specific models, and in vivo animal models will be essential to fully evaluate the systemic safety, pharmacokinetic profile, and therapeutic efficacy of this SQLE-targeted combination regimen across the complete spectrum of osteosarcoma.

Importantly, evaluating these therapeutic limitations requires a deeper understanding of the broader microenvironmental landscape. Recent single-cell RNA sequencing (scRNA-seq) studies have revealed the complex cellular heterogeneity within osteosarcoma tissues, comprising at least nine distinct cell populations [46]. Beyond its direct cytotoxic effects on osteoblastic osteosarcoma cells, the proposed mechanism of terbinafine should be evaluated in the context of the TME. Specifically, key DEGs such as mucin 1-cell surface associated (*MUC1*), collagen type XIII alpha 1 chain (*COL13A1*), jagged canonical notch ligand 2 (*JAG2*), and kazal type serine peptidase inhibitor domain 1 (*KAZALD1*), which have been previously implicated in cancer progression, are predominantly expressed in non-malignant stromal components, including endothelial cells (ECs) and carcinoma-associated fibroblasts (CAFs). Literature further underscores that ECs facilitate tumor vascularization, whereas CAFs drive solid tumor growth and metastasis [47]. Terbinafine may exert a multi-targeted therapeutic outcome by suppressing EC-mediated angiogenesis and disrupting CAF-driven extracellular matrix remodeling.

Considering the critical role of abnormal lipid crosstalk within the osteosarcoma microenvironment—such as CAF-derived fatty acid utilization, lipid-induced dendritic cell dysfunction, and lipid-driven M2/M3 macrophage polarization [48]—targeting lipid pathways presents a promising therapeutic strategy. In this context, terbinafine, a known squalene epoxidase inhibitor that disrupts lipid biosynthesis, may exert multifaceted effects in the TME. Beyond suppressing osteosarcoma cell proliferation, terbinafine could potentially disrupt lipid transfer between CAFs and tumor cells, alleviate lipid accumulation in dendritic cells to restore anti-tumor immunity, and attenuate pro-tumorigenic macrophage polarization. Future studies exploring the precise impact of terbinafine on these lipid-mediated intercellular communications will be highly informative.

## 5. Conclusions

In conclusion, *SQLE* was nominated as an exploratory candidate target, as it did not achieve FDR significance in this clinical transcriptomic cohort. Nevertheless, clinical survival analysis using the TCGA dataset confirmed that high *SQLE* expression serves as a poor prognostic marker associated with reduced sarcoma patient survival. Functional target validation in highly chemoresistant SaOS-2 cells exhibiting the highest baseline *SQLE* levels revealed that SQLE inhibition via terbinafine effectively sensitizes cells to doxorubicin, driving cell death through ferroptosis and partial apoptosis while simultaneously eliminating polyploid populations. Furthermore, target blockade of SQLE with terbinafine reduced metastatic progression by suppressing cell migration and invasion, concomitantly downregulating key mesenchymal and extracellular matrix-degrading markers, including vimentin, MT1-MMP, and MMP-2. Mechanistically, these anti-metastatic effects were driven by the coordinated suppression of the downstream PI3K/AKT/mTOR and JNK/MAPK signaling cascades. Collectively, while transcriptomic findings remain exploratory, these in vitro results provide valuable proof-of-concept evidence supporting further investigation of targeting SQLE with terbinafine as a potential therapeutic strategy in high-grade osteosarcoma.

## Data Availability

The original data presented in the study are openly available in the NCBI Gene Expression Omnibus (GEO) repository under accession number GSE342958.

## Supplementary Materials

The following supporting information can be downloaded at: https://www.mdpi.com/article/10.3390/antiox15091122/s1, Figure S1: Transcriptomic profiling and functional network analysis of chemotherapeutic response in osteosarcoma; Figure S2: Protein–protein interaction (PPI) network and hub gene identification of significantly upregulated DEGs; Table S1: Genes with a |log_2_FC| ≥ 1.4 and a *p*-value < 0.05; Table S2: Metadata; Table S3: Genes with a |log_2_FC| ≥ 1.4 and a p_adj_ < 0.05.

## Abbreviations

The following abbreviations are used in this manuscript:

SQLE	HMG-CoA reductase
DEGs	Differentially expressed genes
GO	Gene ontology
MMP	Mitochondrial membrane potential
EMT	Epithelial–mesenchymal transition
AKT	Protein Kinase B
mTOR	Mechanistic target of rapamycin
GSK3	Glycogen synthase kinase-3
ERK	Extracellular signal-regulated kinase
JNK	c-Jun N-terminal kinase
