# Supplementary material for: Targeting SQLE-Mediated Cholesterol Metabolism to Promote Oxidative Stress and Attenuate Drug Resistance in Osteosarcoma
Source: Antioxidants (Basel). 2026 Sep 4;15(9):1122. doi: 10.3390/antiox15091122 (PMC13603089; doi:10.3390/antiox15091122)
Supplement: Supplementary file 1 [file antioxidants-15-01122-s001.zip › Supplement Figures-03092026.pdf]

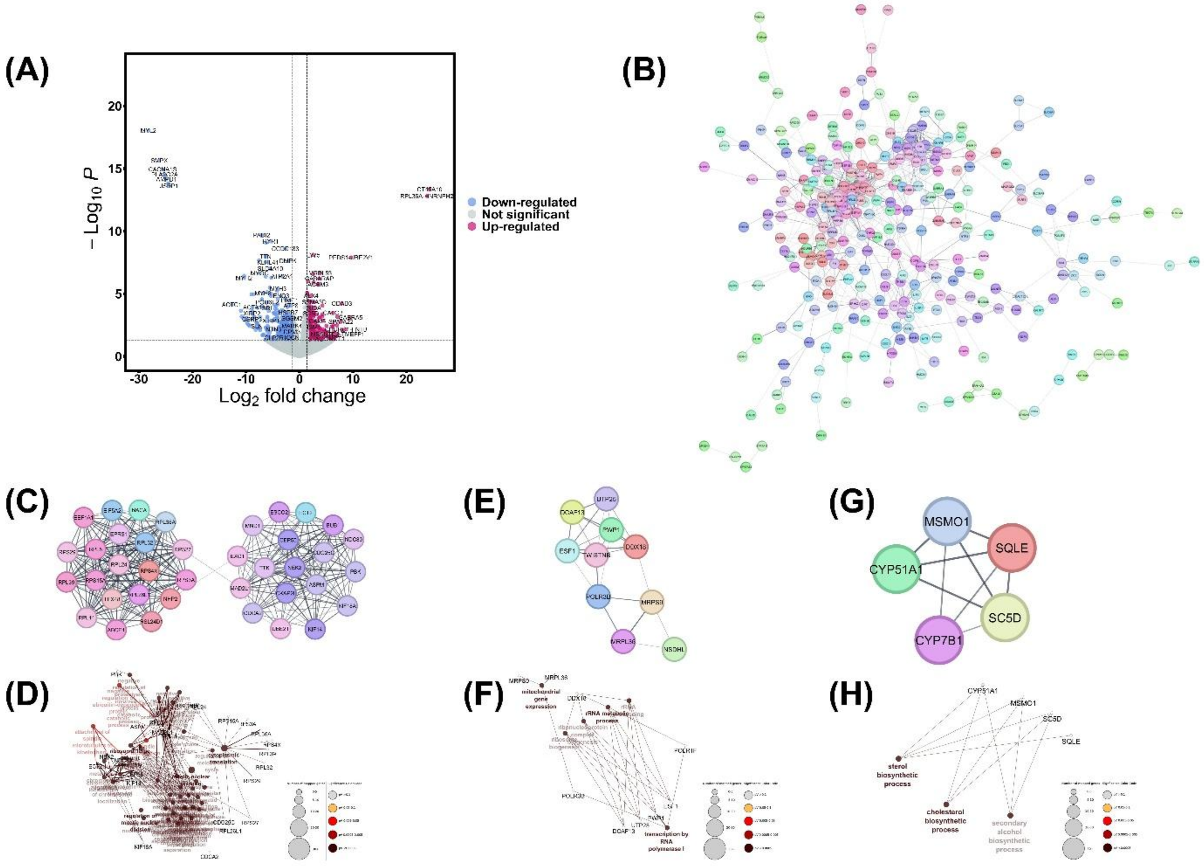

Figure S1. Transcriptomic profiling and functional network analysis of chemotherapeutic response in osteosarcoma.

(A) Volcano plot illustrating DEGs between good and poor chemotherapy response groups. Pink and blue dots represent significantly 446 upregulated and 373 downregulated genes, respectively.

(B) Protein-protein interaction (PPI) network of the identified DEGs constructed using STRING and Cytoscape.

(C) Subnetwork-cluster 1 hub genes identified by MCODE analysis (MCODE score = 18.05).

(D) ClueGO functional enrichment and pathway analysis of the hub genes in panel (C).

(E) Subnetwork-cluster 2 hub genes identified by MCODE analysis (MCODE score = 5.33).

(F) ClueGO functional enrichment and pathway analysis of the hub genes in panel (E).

(G) Subnetwork-cluster 3 hub genes identified by MCODE analysis (MCODE score = 4.50).

(H) ClueGO functional enrichment and pathway analysis of the hub genes in panel (G).

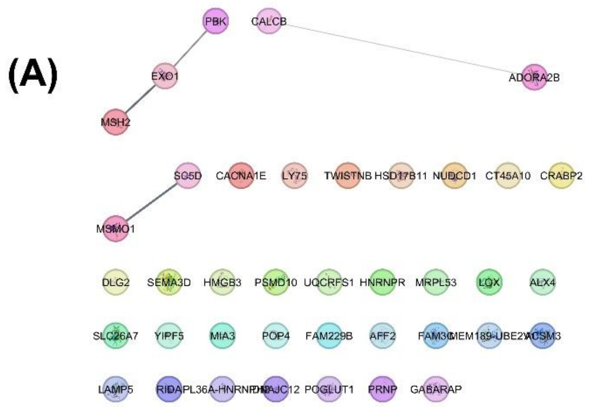

Figure S2. Protein-protein interaction (PPI) network and hub gene identification of significantly upregulated DEGs.

(A) PPI network constructed using STRING for the significantly upregulated genes meeting the criteria of padj < 0.05 and log2FC ≥ 1.4. Nodes represent proteins and edges represent functional interactions.

(B) Top candidate hub genes identified via cytoHubba analysis using the Maximal Clique Centrality (MCC) algorithm. Node colors range from red (highest rank) to orange and yellow, reflecting their topological importance within the network.
